# Semiclassical Theory of Stepped Electrodes and Step Bunching

**DOI:** 10.1021/jacs.6c10687

**Published:** 2026-07-14

**Authors:** Zengming Zhang, Michael Eikerling, Jun Huang

**Affiliations:** † Institute of Energy Technologies, IET-3: Theory and Computation of Energy Materials, 28334 Forschungszentrum Jülich GmbH, Jülich 52425, Germany; ‡ Faculty of Georesources and Materials Engineering, RWTH Aachen University, Aachen 52062, Germany

## Abstract

Atomic-scale steps markedly influence electrochemical activity and stability and exhibit structural instability under electrochemical conditions. Yet the microscopic mechanisms that cause these behaviors remain largely unclear. Herein, we study the microstructure and thermodynamics of the electrical double layer at stepped electrodes, using the semiclassical density-potential functional theory. The theory captures trends observed in experiments regarding the differential capacitance and the potential of zero free charge (PZFC) with step density for stepped Au and Ag . Departing from the case of flat electrodes, the PZFC deviates from the potential of minimum capacitance at stepped electrodes, necessitating local PZFCs to describe heterogeneous surface charging conditions. Furthermore, linking step-induced PZFC shifts to changes of the surface tension, the theory predicts that step bunching is thermodynamically driven at more positive electrode potentials and sensitive to the electrolyte composition.

## Graphical abstract

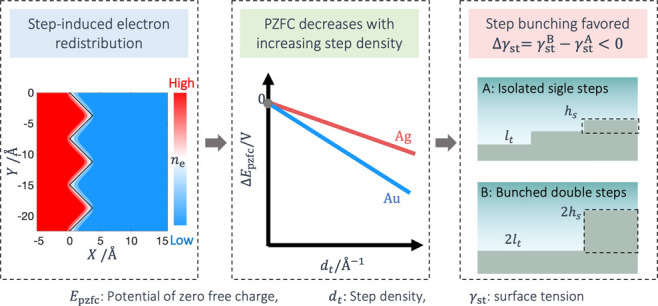

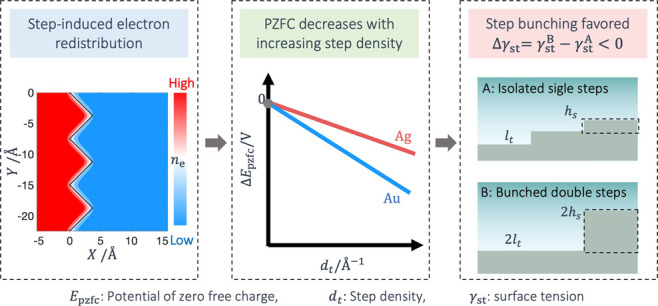

## 1 Introduction

Atomic steps are among the most catalytically active motifs on metal electrodes and play a decisive role in determining the activity, selectivity, and stability in electrocatalytic systems.^1–8^ Experimental studies on stepped single-crystal electrodes (SSCEs) have shown that reaction rates for key processes, such as hydrogen evolution,^5,9–11^ oxygen reduction,^12–14^ and small-molecule oxidation,^15–17^ can vary by orders of magnitude with step density and step geometry. These observations establish step sites as dominant contributors to electrocatalytic performance.

SSCEs provide a controlled model system in which the step density and step orientation can be tuned independently while maintaining atomic precision. SSCE studies have revealed that step sites exhibit distinct adsorption energetics,^18–22^ altered local electronic structure,^6,19^ and characteristic work function shifts relative to terrace atoms,^23–27^ leading to measurable changes in voltammetric features and differential double layer capacitance, *C*_dl_. Theoretical studies,^28–32^ including density functional theory and *ab initio* molecular dynamics simulations, have revealed step-induced charge redistribution, modified water structure, and dipole formation at step edges.

The structural stability of stepped electrodes under electrochemical conditions remains much less explored. Recent experiments have shown that the potential of zero free charge (PZFC) and the catalytic activity deviate from simple linear scaling relations with step density, when terraces become sufficiently narrow, signaling the emergence of collective electrostatic and mechanical effects at high step densities.^13,33^ These deviations have been associated with surface reconstruction and step bunching.^4,34–37^ While step bunching is well understood for vicinal surfaces in vacuum, as a consequence of surface free-energy minimization and facet formation,^34,38–40^ it remains unresolved whether analogous energetically driven processes operate at electrified metal-solution interfaces, where electrostatics, solvation, and ionic screening play central roles.

Herein, we study the EDL at stepped electrodes and its structural stability by developing a thermodynamic description of stepped metal-solution interfaces under constant-potential conditions, using a semiclassical density-potential functional theoretical (DPFT) model.^41,42^ Applying this approach to SSCEs of stepped Au and Ag in weakly adsorbing electrolytes, we quantify how the step density modifies the EDL, the PZFC, and the surface tension. The surface tension decreases at high step densities and elevated electrode potentials, constituting the thermodynamic driving force for step bunching.

## 2 Theoretical and Computational Methods

The stepped metal-solution interfaces were modeled under constant-potential conditions using DPFT.^41,42^ The framework couples an orbital-free description of metal valence electrons with a classical statistical-field treatment of the electrolyte, allowing the electronic density, ionic distributions, and electrostatic potential to be determined self-consistently. The model was applied to stepped Au and Ag single-crystal electrodes with systematically varied step densities to evaluate the EDL structure, PZFC, *C*_dl_, and surface tension. Full details of the theoretical formulation, model parameters, boundary conditions, and numerical implementation are provided in the Supporting Information.^43^

## 3 Results

### 3.1 Parametrization of the DPFT Model

We begin by calibrating the DPFT model against experimental *C*_dl_ data for flat single-crystal Au^44–46^ and Ag^47–49^ electrodes in weakly adsorbing electrolytes. An extended comparison between model and experiment is shown in Figure S1 of the Supporting Information.^43^ The model captures the behavior of experimental *C*_dl_ curves across different electrolyte compositions and concentrations for both electrodes, with reasonable parametrization, as discussed in the Supplementary Note S1. Parametrized at flat single-crystal electrodes, it is extended to study the influence of steps. Figure 1(a) shows a schematic diagram of the SSCE, characterized by a terrace width *l*_*t*_ and a step height *h*_*s*_. The step density is defined as *d*_*t*_ = *l*_*t*_^–1^. Periodic boundary conditions are used for the rotated geometry, as shown in the upper-middle panel of Figure 1(a). The flat electrode corresponds to the limiting case of an infinite terrace width (*l*_t_ *→* ∞), as illustrated in Figure 1(b).

**Figure 1:**
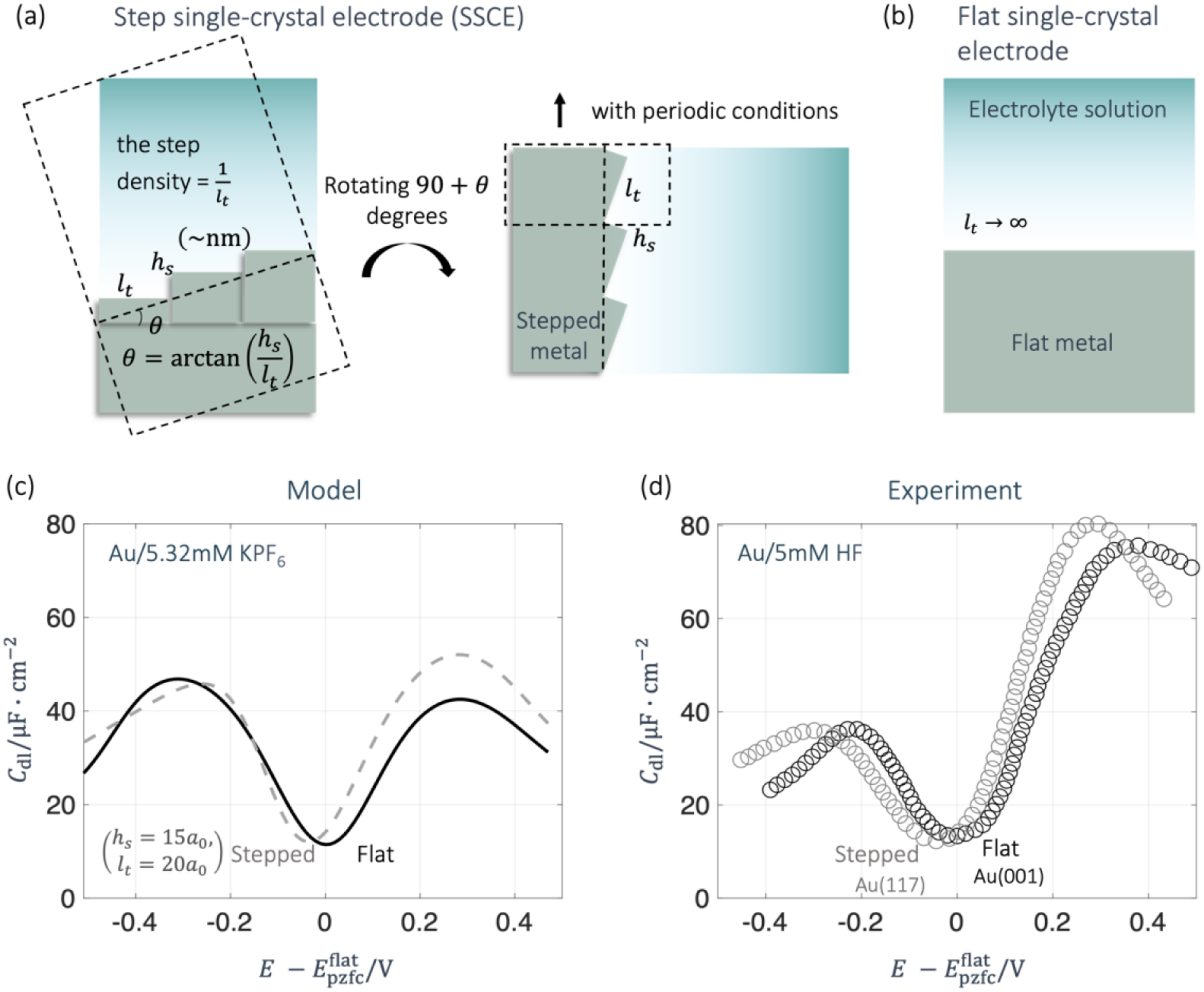
Electrical double layers at stepped single-crystal electrode (SSCE)-solution interfaces. (a) Schematic of a SSCE with terrace width *l*_*t*_ and step height *h*_*s*_; the step density is defined as *d*_*t*_ = *l*_*t*_^–1^. The upper-middle panel shows the periodic modeling geometry used in this work. (b) Schematic of a flat single-crystal electrode, corresponding to the limiting case of *l*_*t*_ → ∞. Comparison of *C*_dl_ between flat and stepped electrodes obtained from (c) the DPFT model and (d) experiments. Model results are shown for *h_s_* = 15*a*_0_ and *l*_*t*_ = 20*a*_0_ with Bohr radius *a*_0_ = 0.529 Å. Experimental data are taken from Beltramo et al.^35^ Electrode potentials are referenced to the PZFC of the flat electrode.

Without changing model parameters other than the electrode geometry, the model captures the step-induced negative shift of the PZFCs, observed experimentally on Au^35^ and Ag^36^, as shown in Figure 1(c) and (d) for the example of an Au SSCE. Originating from a physical description of metal electrons under constant potential conditions, the capability to capture PZFC changes constitutes an advantage of the DPFT model compared to classical EDL models, jellium models,^50,51^ and more recent constant-potential molecular dynamics simulations.^52–56^ Discrepancies are observed in the magnitude of anodic *C*_dl_ peaks, which are primarily attributed to specific adsorption of fluoride ions. Previous experimental and theoretical studies have shown that weak but non-negligible specific adsorption of F^–^ ions even on Au surfaces,^57–59^ which are not considered in this work. Specific adsorption of ions contributes a pseudocapacitance to the measured “capacitance”, elevating anodic capacitance peaks.^58,60,61^

### 3.2 Step-Density Dependence of the PZFC

Extending beyond the specific example shown in Figure 1, we analyze the dependence of the PZFC on step density for stepped Au and Ag electrodes in different electrolytes. In the literature, experimentally *E*_pzfc_ was determined usually as the potential of minimum capacitance (PMC), *E*_pmc_, in dilute electrolyte solutions. The model reveals that *E*_pzfc_ differs from *E*_pmc_ at stepped electrodes by an amount of ∼8 mV. This deviation is intrinsic to the heterogeneous microstructure of the EDL at stepped electrodes, as to be discussed in the next section. Therefore, the model-based *E*_pzfc_ is taken, per definition, as the potential, at which the surface free charge density is zero. The shift in PZFC relative to the value for flat single-crystal electrodes, as a function of step density is denoted as Δ*E*_pzfc_(*d*_*t*_). Figure S2 in the Supporting Information displays results for Δ*E*_pzfc_(*d_t_*) at various values of the step height *h*_*s*_.^43^ In general, increasing the step density and the step height enhances the electron redistribution between the top and bottom of the step site, leading to larger shifts in Δ*E*_pzfc_, as shown in Figure S3. In Figure 2, we compare model predictions, using the model parameters obtained at flat electrodes, with experimental data, using *h_s_* = 15*a*_0_ = 7.9 Å for stepped Ag and *h*_*s*_ = 10*a*_0_ = 5.29 Å for stepped Au electrodes. Linear fits are used to quantify the trends.

**Figure 2:**
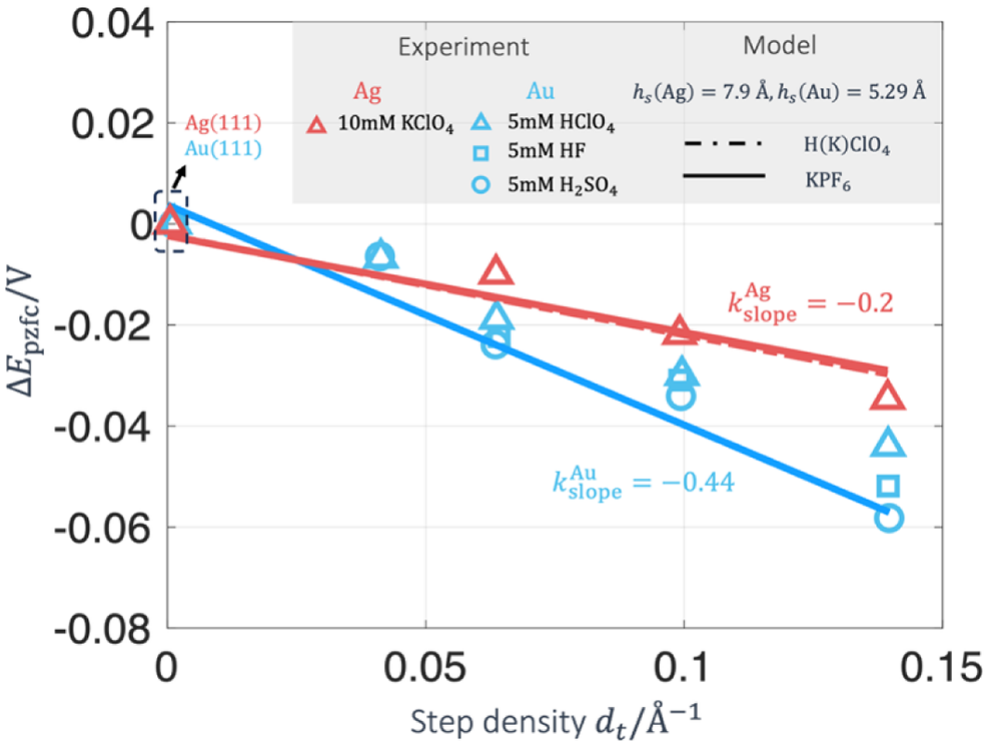
Step-density dependence of the PZFC at stepped electrodes relative to that at flat single-crystal electrodes, Δ*E*_pzfc_(*d*_*t*_) in aqueous electrolytes. The model predictions obtained with model parameters calibrated at flat electrodes (solid line) are compared with experimental data (symbols) taken from refs 35,36 . The experimental data were obtained on vicinal Au(001) and Ag(001) single-crystal electrodes with controlled step densities, where capacitance–potential curves were measured by impedance spectroscopy and the PZFC values were extracted from the capacitance minima. Model results are shown for a step height *h*_*s*_ = 7.9 Å for stepped Ag and *h*_*s*_ = 5.29 Å for stepped Au.

The model reproduces the experimentally observed linear decreases of Δ*E*_pzfc_(*d_t_*) with increasing step density for both stepped Au and Ag electrodes across different electrolytes.^35,36^ From the fitted slopes, $k_{\mathrm{step}}^{\mathrm{Ag}}=-0.2\;V\cdot Å$ and $k_{\mathrm{step}}^{\mathrm{Au}}=-0.44\;V\cdot Å$ , the step-edge dipole moment *p*_step_ can be extracted using the relation,^4^

1 $$\Delta E_{\mathrm{pzfc}}=-\frac{p_{\mathrm{step}}}{\epsilon_{\mathrm{IHP}}d_{\mathrm{atom}}l_{t}}$$

where ϵ_IHP_ is the permittivity at the inner Helmholtz plane, taken 6*ϵ*_0_ ^62^ and *d*_atom_ = 2.89 Å is the spacing between densely packed atomic rows along [110].^35^ The resulting step dipole moments are $p_{\mathrm{step}}^{\mathrm{Ag}}\approx 1.92\times 10^{-2}{\;e}_{0}\cdot Å$ and $p_{\mathrm{step}}^{\mathrm{Au}}\approx 4.22\times 10^{-2}{\;e}_{0}\cdot Å$ . These values are larger than values obtained fromprevious estimates, $p_{\mathrm{step}}^{\mathrm{Ag}}\approx \left(3.5\pm 0.5\right)\times 10^{-3}{\;e}_{0}\cdot Å$ for Ag in perchlorate electrolytes^36^ and $p_{\mathrm{step}}^{\mathrm{Au}}\approx \left(5-7\right)\times 10^{-3}{\;e}_{0}\cdot Å$ for Au depending on electrolyte composition,^35^ because they used ϵ_IHP_ = ϵ_0_ in eq 1 while we use a more realistic higher value. The results indicate that the step-induced PZFC shifts at Ag and Au SSCEs is mainly caused by the intrinsic redistribution of valence electrons at step edges, which can adequately be captured by the DPFT model. When Au(110) is treated as the limiting high-step-density member of a stepped (111) × (111)-type series, its geometric step density is *d*_*t*_ ≈ 0.30 Å^*–*1^. The linear trend between Δ*E*_pzfc_ and *d*_*t*_ yields *E*_pzfc_ ≈ 0.42 V_SHE_ for Au(110), whereas the measured value is 0.30 V_SHE_ (see Figure S1­(e)). While the theory captures the sign of change correctly, the underesimated magnitude of change suggests that Au(110) is beyond the dilute-vicinal regime and that reconstruction or strong step–step interactions contribute to the additional PZFC decrease.

We further examined stepped Pt electrodes in Figure S4.^43^ In the initial DPFT formulation, the model qualitatively reproduces the decrease of the potential of zero total charge (PZTC) with step density but significantly underestimates its magnitude. Experiments show larger negative shifts that emerge in the hydrogen adsorption region, where faradaic charge and adsorption pseudocapacitance dominate the interfacial response.^18–20,22^ As demonstrated by Gómez et al., the measured PZTC of stepped Pt electrodes reflects a convolution of intrinsic step dipoles and step-specific chemisorption, rather than purely electrostatic effects.^25^ To account for this contribution, we extended the DPFT framework by incorporating chemisorption effects.^63,64^ With this extension, the model reproduces the experimentally observed step-density dependence of the PZTC more accurately, yielding significantly improved agreement with measurements (see Figure S4 ^43^). These results highlight the important role of adsorption processes in determining the electrochemical response of Pt step edges and demonstrate that their inclusion is essential for quantitatively describing Pt electrodes within the DPFT framework.

### 3.3 Heterogeneous EDL and Its Characteristic Potentials

The deviation between the PZFC and the PMC points to heterogeneity of the EDL at stepped metal-solution interfaces, which require local PZFCs to properly characterize the heterogeneous surface charging conditions.

Figure 3(a–d) shows the EDL microstructure at the PZFC for a representative symmetrically stepped Au electrodes with *l*_*t*_ = *h*_*s*_ = 10*a*_0_ = 5.29 Å in KPF_6_ aqueous solution. Spillover of electron density preferentially occurs into the step valleys from the step peaks, known as the Smoluchowski effect.^65,66^ Therefore, the electric potential is more positive near step peaks [Figure 3(a, b)]. This electronic redistribution produces a heterogeneous ionic environment in the EDL at the PZFC, with excess cations in step valleys and excess anions near step peaks [Figure 3(c, d)].

**Figure 3:**
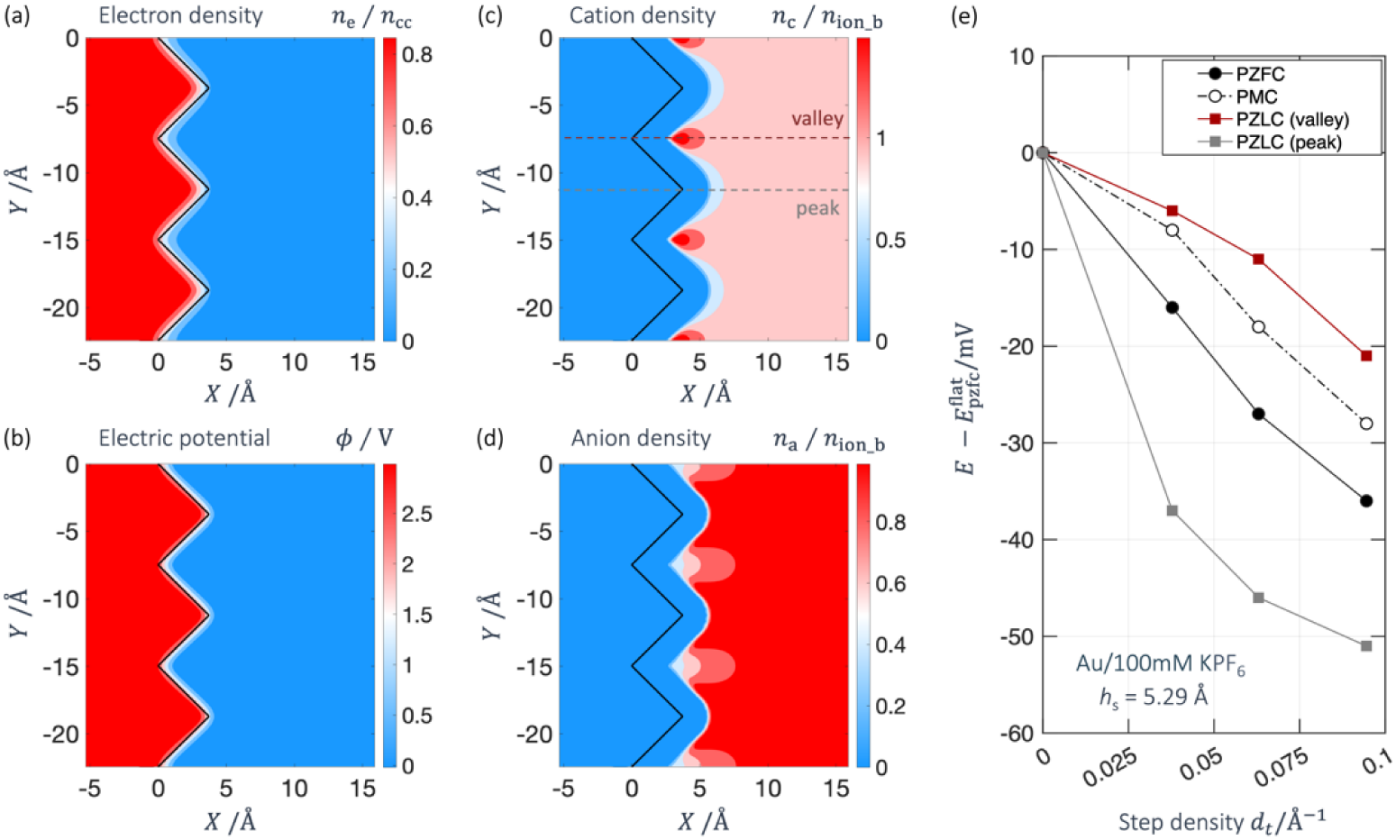
Heterogeneities of the EDL at stepped Au (*l*_*t*_ = *h*_*s*_ = 10*a*_0_ = 5.29 Å) in aqueous KPF solution at the PZFC. Spatial distribution of (a) dimensionless electron density ${\mathrm{n̅}}_{e}$ relative to the metal atomic-core charges *n*_cc_, (b) electric potential *ϕ*, (c) dimensionless cation density ${\mathrm{n̅}}_{c}$ , and (d) dimensionless anion density ${\mathrm{n̅}}_{a}$ , normalized by the bulk ion concentration *n*_ion_b_. (e) Step-density dependence of the global PZFC, the potential of minimum capacitance (PMC), and the local potential of zero charge at step valley [PZLC­(valley)] and peak [PZLC­(peak)], all referenced to the PZFC of the flat single-crystal electrode. Black lines indicate the metallic surface profile in (a)–(d).

The heterogeneous EDL implies that different surface regions reach local charge neutrality at different electrode potentials, similar to the EDL at supported nanoparticles as studied by Zhang et al.^67^ Figure 3(e) compares the step-density dependence of the global PZFC, the PMC, and the local PZFCs (PZLC) at the step valley and peak, defined as the potentials, at which the net ionic charge integrated along the horizontal direction across the respective valley or peak region vanishes [see dashed lines in Figure 3(c)]. All characteristic potentials decrease monotonically with the step density *d*_*t*_. However, the PMC is approximately 8 mV more positive than the global PZFC. The PZLC at the step valley is relatively more positive, more strongly repelling excess cations, whereas the PZLC at the step peak is more negative, thus more strongly repelling excess anions. The global PZFC corresponds to a vanishing net surface free charge, whereas the PMC reflects the minimum of the differential electrostatic response. Because the stepped interface exhibits spatially varying electric fields and screening environments, the capacitance minimum is governed by the balance of local valley and peak responses rather than by global charge neutrality. Hence, the PZFC differs from the PMC, which has been also observed for rough electrodes,^68^ polycrystalline electrodes^69^ and EDLs at supported nanoparticles.^67^ This separation is relevant for interpreting structure-sensitive electrochemical behavior because the PMC cannot generally be used as a direct proxy for zero surface charge on stepped electrodes. Even at the global PZFC, valley and peak regions can remain locally polarized, creating distinct local electric fields experienced by adsorbates and reacting species. Thus, the step-density-dependent shifts of PZFC, PMC, and PZLC connect microscopic EDL heterogeneity with capacitance, surface free energy, adsorption energetics, and structural stability.

### 3.4 Step-density Dependence of Surface Tension

Inspired by the specific model calculations, we present a conceptual analysis of the dependence of the surface tension on the step density, which is thermodynamically equivalent to an excess grand-canonical potential density (see Figure S5 ^43^). Based on the definition of electrocapillarity, the surface tension can be expressed, up to a constant, as the integral of the surface charge density, *σ*_free_, over the electrode potential, as described by the Lippmann equation.^70–72^ In this article, we consider the relative surface tension, Δ*γ*_*st*_, referenced to the PZFC, written as,

2 $$\Delta \gamma_{\mathrm{st}}=\gamma_{\mathrm{st}}-\gamma_{\mathrm{st}}^{0}=-\int_{E_{\mathrm{pzfc}}}^{E_{M}}\sigma_{\mathrm{free}}dE$$

where $\gamma_{\mathrm{st}}^{0}$ denotes the surface tension at the PZFC. Differentiation of Δ*γ*_st_ with respect to the step density *d*_*t*_ at fixed electrode potential yields,

3 $$\begin{array}{ll} {\left(\frac{\partial \Delta \gamma_{\mathrm{st}}}{\partial d_{t}}\right)}_{E} & =-\frac{\partial }{\partial d_{t}}\int_{E_{\mathrm{pzfc}}}^{E_{M}}\sigma_{\mathrm{free}}dE\\ & =\sigma_{\mathrm{free}}\frac{\partial E_{\mathrm{pzfc}}}{\partial d_{t}}-\int_{E_{\mathrm{pzfc}}}^{E_{M}}\frac{\partial \sigma_{\mathrm{free}}}{\partial d_{t}}dE \end{array}$$

As shown in Figure S6 of the Supporting Information,^43^ the explicit dependence of the surface charge density on the step density is weak over the range considered here, consistent with experimental observations for weakly adsorbing electrolytes.^35,36^ A quantitative evaluation of eq 3 using the model yields $\sigma_{\mathrm{free}}\frac{\partial E_{\mathrm{pzfc}}}{\partial d_{t}}∼10^{-1}\frac{J}{m^{2}}a_{0}$ , whereas $\int_{E_{\mathrm{pzfc}}}^{E_{M}}\frac{\partial \sigma_{\mathrm{free}}}{\partial d_{t}}dE∼10^{-3}\frac{J}{m^{2}}a_{0}$ . The latter is therefore negligible to leading order, and eq 3 is approximated well by,

4 $${\left(\frac{\partial \Delta \gamma_{\mathrm{st}}}{\partial d_{t}}\right)}_{E}\approx \sigma_{\mathrm{free}}\frac{\partial E_{\mathrm{pzfc}}}{\partial d_{t}}$$

Equation 4 reveals that the dependence of the surface tension on the step density is governed by two physically distinct contributions: *σ*_free_, which is controlled by the electrode potential and electrolyte properties (e.g., ionic concentration, see Figure S7 of the Supporting Information,^43^ and composition), and $\frac{\partial E_{\mathrm{pzfc}}}{\partial d_{t}}$ , which is an electrode-specific property. As demonstrated in Figure 2, *E*_pzfc_ decreases approximately linearly with increasing step density, such that $\frac{\partial E_{\mathrm{pzfc}}}{\partial d_{t}}$ is negative and nearly constant for a given metal surface. As a result, the relative surface tension Δ*γ*_st_ varies approximately linearly with step density, as illustrated schematically in Figure 4(a). Stepped Au electrodes exhibit a larger magnitude of this slope due to a greater sensitivity of the PZFC to step density. A change of Δ*γ*_st_ with varying step density implies that configurations with redistributed step density can lower the total interfacial free energy, which constitutes the thermodynamic driving force of step bunching.

**Figure 4:**
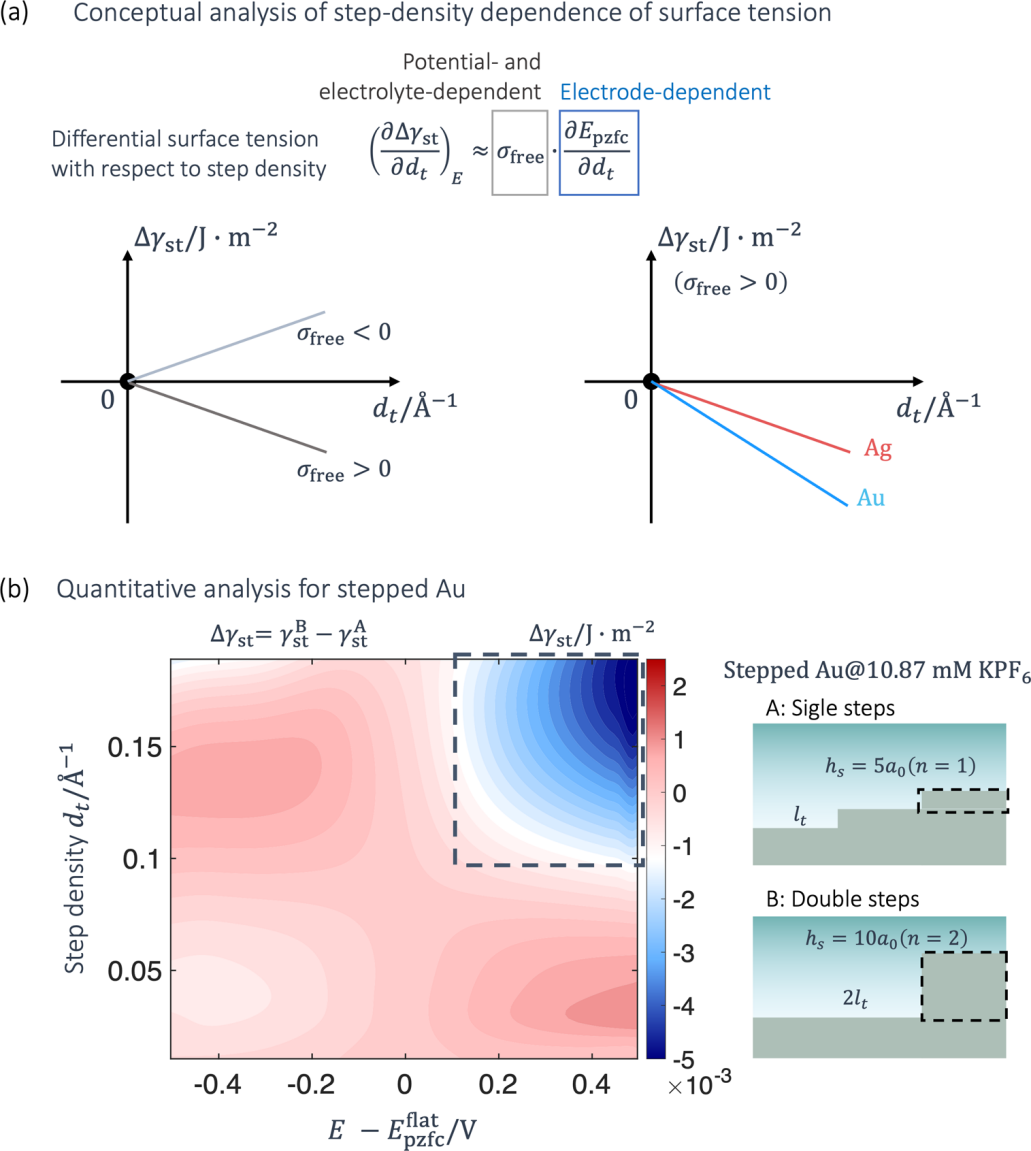
(a) Conceptual illustration of the step density dependence of the relative surface tension Δ*γ*_st_(*d*_*t*_), highlighting its separation into a potential- and electrolyte-dependent surface charge contribution *σ*_free_ and an electrode-dependent PZFC shift, $\frac{\partial E_{\mathrm{pzfc}}}{\partial d_{t}}$ . (b) Phase map of the change in surface tension, $\Delta \gamma_{\mathrm{st}}=\gamma_{\mathrm{st}}^{B}-\gamma_{\mathrm{st}}^{A}$ , between double step (B) and single-step (A) configurations for Au in 10 mM KPF_6_ solution, as a function of step density *d*_*t*_ and electrode potential relative to the PZFC of the flat surface. Negative values indicate a thermodynamic driving force for step bunching. Insets schematically illustrate the single- and double-step geometries.

### 3.5 Quantitative Analysis of Step Bunching for Stepped Au

As a concrete example, we consider stepped Au electrodes in 10 mM KPF_6_ aqueous solution. Figure 4(b) presents a phase diagram of the change in relative surface tension,

$$\Delta \gamma_{\mathrm{st}}=\gamma_{\mathrm{st}}^{B}-\gamma_{\mathrm{st}}^{A}$$ 5

between double-step (B) and single-step (A) configurations, as schematically shown in the inset. A pronounced region with Δ*γ*_st_ < 0 emerges at high step density and positive electrode potential, indicating that step bunching is thermodynamically favorable under these conditions. In contrast with the case in section 3.4 with a fixed *h*_s_, the present case involves changes in both *d*_t_ and *h*_s_ simultaneously. Therefore, Eq.(4) is modified as,

6 $${\left(\frac{\left(\partial \Delta \gamma_{st}\right)}{\left(\partial d_{t}\right)}\right)}_{E}=\sigma_{free}\left(\frac{{\partial E}_{pzfc}}{{\partial d}_{t}}+\frac{{\partial E}_{pzfc}}{{\partial h}_{s}}\frac{{\partial h}_{s}}{{\partial d}_{t}}\right)$$

where the second term accounts for the accompanying change in step height. While $\frac{{\partial E}_{pzfc}}{{\partial d}_{t}}$ < 0, the second term is positive as $\frac{{\partial h}_{s}}{{\partial d}_{t}}$ < 0 and $\frac{{\partial E}_{pzfc}}{{\partial h}_{s}}$ < 0 as shown in Figure S2. When the second term dominates over the first term in the right hand side of eq 6, step bunching with decreasing *d_t_* but increasing *h_s_* will decrease Δγ_st_ at σ_free_ > 0; this is why the right upper corner of Figure 4(b) is negative.

Ibach and Schmickler developed a phenomenological theory of step instability,^34^ which revealed that the variation of PZFC with step density can drive phase separation of stepped electrodes into step-rich and flat regions. More recently, EC-STM measurements by Koper and co-workers^37^ demonstrated that Pt vicinals with high step density spontaneously form bunched structures. Our analysis provides a self-consistent microscopic description of the EDL at stepped electrodes under constant-potential conditions, enabling direct computation of the surface tension as a function of electrode potential and step density. Improving over the phenomenological theory of Ibach and Schmickler,^34^ the present framework captures spatially resolved charge redistribution and capacitance variations, thereby quantifying the thermodynamic competition between electrostatic driving forces and step–step interactions. The resulting phase diagram identifies the potential window and electrolyte conditions under which step bunching becomes favorable, establishing a direct link between EDL microstructure and morphological instability.

## 4 Conclusion

In summary, we have employed a semiclassical, hybrid density-potential functional theory (DPFT) to model the EDL at stepped metal-solution interfaces under constant-potential conditions. The theory reproduces and provides microscopic insights into experimental trends in differential capacitance and the nearly linear decrease of the potential of zero free charge (PZFC) with step density for stepped Au and Ag electrodes. Beyond global quantities like PZFC, local potentials of zero charge are defined to describe the essential heterogeneities of the EDL at stepped electrodes. Furthermore, by connecting step-induced shifts in the PZFC to changes in surface tension, we demonstrate that step bunching at high step densities is thermodynamically driven at more positive electrode potentials and modulated by electrolyte composition. In addition, chemisorption effects need to be augmented into the DPFT model to provide an adequate description of stepped Platinum electrodes.

## Supplementary Material



## Notes

Calibrating the DPFT model at flat single-crystal electrodes; effects of step density and height on the change in the PZFC; effects of step density and height on the electron spillover at PZFC; step density dependence of the PZFC on Pt electrode; equivalence of surface tension and excess grand-canonical potential density; effect of step density on the surface charge density; ionic concentration effect on step bunching; theoretical and computational methods include variational analysis, numerical implementation and calculation of thermodynamic properties; Table S1: basic model parameters; Table S2: parameters in the DPFT model; and Table S3: parameters in the DPFT_chem model (PDF)
