# Supplementary material for: Semiclassical Theory of Stepped Electrodes and Step Bunching
Source: J Am Chem Soc. 2026 Jul 14;148(29):31510–7. doi: 10.1021/jacs.6c10687 (PMC13426266; doi:10.1021/jacs.6c10687)
Supplement: Supplementary file 1 [file ja6c10687_si_001.pdf]

Supporting Information to

Semiclassical Theory of Stepped Electrodes and Step Bunching

Zengming Zhang[1,2], Michael Eikerling[1,2], Jun Huang[1,2,*]

[1] *Institute of Energy Technologies, IET-3: Theory and Computation of Energy Materials, Forschungszentrum Jülich GmbH, 52425 Jülich, Germany*

[2] *Faculty of Georesources and Materials Engineering, RWTH Aachen University, Aachen 52062, Germany*

* *Corresponding author: ju.huang@fz-juelich.de*

Table of Contents

1. Calibrating the DPFT model at flat single-crystal electrodes **2**

2. Effects of step density and height on the change in the PZFC **4**

3. Effects of step density and height on the electron spillover at PZFC **5**

4. Step density dependence of the PZTC on Pt electrode **6**

5. Equivalence of surface tension and excess grand-canonical potential density **7**

6. Effect of step density on the surface charge density **8**

7. Ionic concentration effect on step bunching **9**

8. Theoretical and Computational Methods **10**

- **Variational analysis** 12
- **Numerical implementation** 14
- **Calculation of thermodynamic properties** 18

Table S1. Basic model parameters **20**

Table S2. Parameters in the DPFT model **21**

Table S3. Parameters in the DPFT_chem model[47,48] **22**

Reference **24**

1. Calibrating the DPFT model at flat single-crystal electrodes

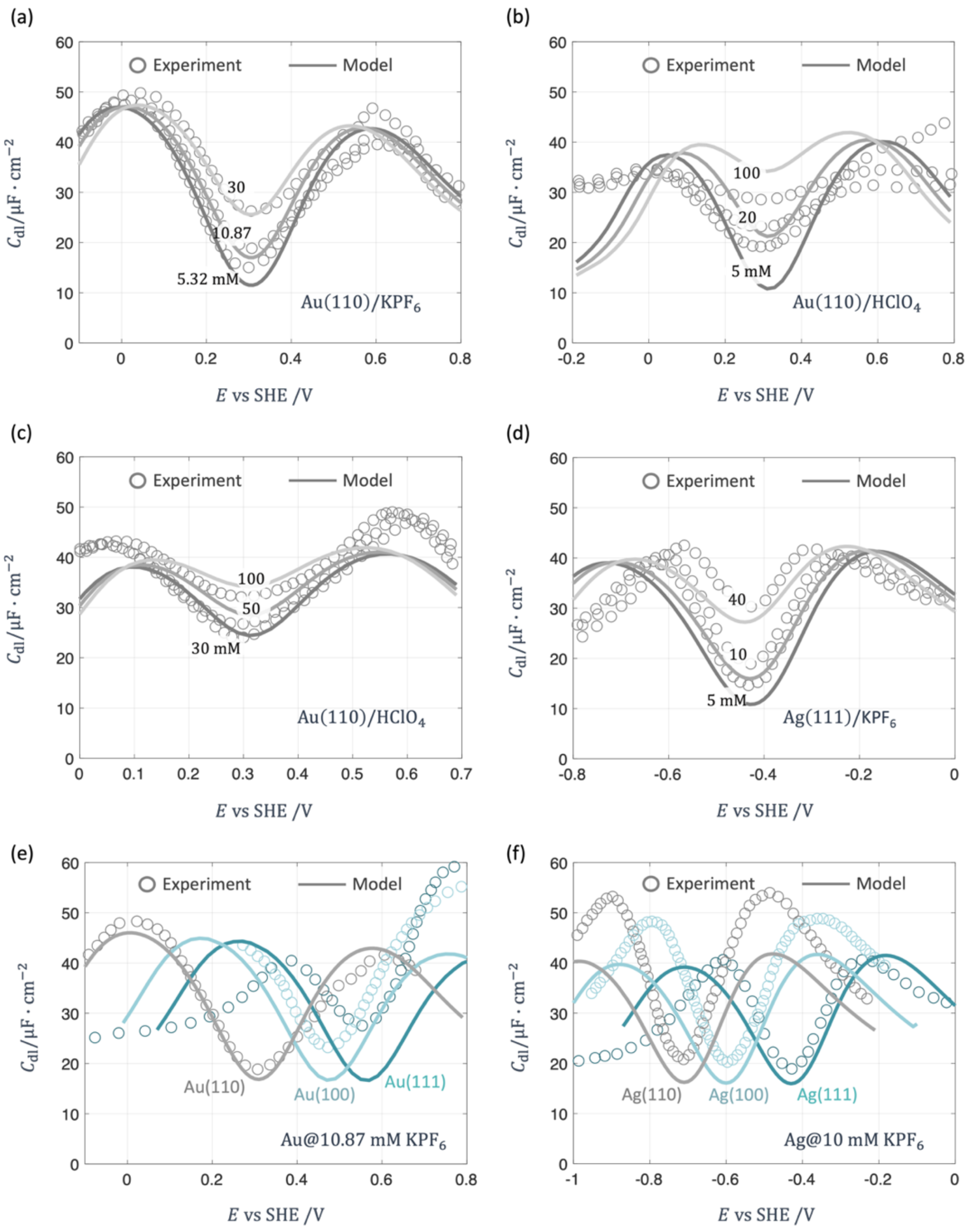

Figure S1. Calibration of the DPFT model (solid lines) with experimental C_{dl} data (circles) for (a) $\mathrm{Au(110)/KPF_6}$ measured by Hamelin et. al.,[1] (b, c) $\mathrm{Au(110)/HClO_4}$ measured by Samec, et. al.,[2] and Hamelin et. al.,[3] respectively, and (d) $\mathrm{Ag(111)/KPF_6}$ measured by Valette, et. al.,[4]. (e) $\mathrm{Au(111, 100, 110)/KPF_6}$ measured by Hamelin et. al.,[1] and (f) $\mathrm{Ag(111, 100, 110)/KPF_6}$ measured by Valette, et. al.[5,6,4]. Calibrated parameters are $v_{\mathrm{cc}}^{0,\mathrm{Au(110)}} = -0.549$ eV, $d_{\mathrm{mK^+(PF_6^-)}}^{\mathrm{Au}} = 1.8$ Å, $d_{\mathrm{mH^+(ClO_4^-)}}^{\mathrm{Au}} = 2.8$ Å, and $v_{\mathrm{cc}}^{0,\mathrm{Ag(111)}} = -0.394$ eV, $d_{\mathrm{mK^+}}^{\mathrm{Ag}} = 4.8$ Å, $d_{\mathrm{mPF_6^-}}^{\mathrm{Ag}} = 1.8$ Å. $v_{\mathrm{cc}}^{0,\mathrm{Au(111)}} = -0.572$ eV, $v_{\mathrm{cc}}^{0,\mathrm{Au(100)}} = -0.564$ eV and $v_{\mathrm{cc}}^{0,\mathrm{Ag(100)}} = -0.363$ eV, $v_{\mathrm{cc}}^{0,\mathrm{Ag(110)}} = -0.343$ eV. The electrode potential is on the SHE scale.

A comparison of C_{dl} between model and experiment is plotted in Figure S1. Experimental data of $\mathrm{Au(110)/KPF_6}$ are measured by Hamelin et. al.,[1] $\mathrm{Au(110)/HClO_4}$ by Samec, et. al.,[2] and Hamelin et. al.,[3] and $\mathrm{Ag(111)/KPF_6}$ by Valette, et. al.,[4]. The calibrated parameters for Au(110) include $v_{\mathrm{cc}}^{0,\mathrm{Au(110)}} = -0.549$ eV, $d_{\mathrm{mK^+(PF_6^-)}}^{\mathrm{Au}} = 1.8$ Å, $d_{\mathrm{mH^+(ClO_4^-)}}^{\mathrm{Au}} = 2.8$ Å. For $\mathrm{Ag(111)/KPF_6}$ system, the calibrated parameters are $v_{\mathrm{cc}}^{0,\mathrm{Ag(111)}} = -0.394$ eV, $d_{\mathrm{mK^+}}^{\mathrm{Ag}} = 4.8$ Å, $d_{\mathrm{mPF_6^-}}^{\mathrm{Ag}} = 1.8$ Å. A full list of model parameters is provided in Tables S1 and S2.

The model-based C_{dl} agrees reasonably well with experiments across different electrolyte compositions and electrodes. For the Au(110) electrode, the use of $\mathrm{HClO_4}$ solution, which exhibits weaker metal-ion short-range interactions than $\mathrm{KPF_6}$, reduces the peak of C_{dl} on both the cathodic and anodic sides, consistent with trends reported in our recent works[7,8]. Furthermore, for the same electrolyte solution, $\mathrm{KPF_6}$, the metal-cation short-range interactions are weaker on Ag(111) than on Au(110). This may be attributed to the fact that Ag(111) exhibits stronger hydrophilicity[9,10], attracting more water molecules to the metal surface and weakening metal-cation interactions. In addition, different crystal facets of the metal electrode exhibit different potential of zero free charges (PZFCs), as shown in Figure S1(e) for Au and Figure S1(f) for Ag electrodes. According to experimental data by Hamelin et. al.[1], the measured E_{pzfc} for Au(111), Au(100) and Au(110) in 10.87mM $\mathrm{KPF_6}$ aqueous solution are 0.55 $\mathrm{V_{SHE}}$, 0.46 $\mathrm{V_{SHE}}$, 0.30 $\mathrm{V_{SHE}}$, respectively. In experiments by Valette, et. al.[5,6,4], the measured E_{pzfc} for Ag(111), Ag(100) and Ag(110) in 10mM $\mathrm{KPF_6}$ aqueous solution are -0.43 $\mathrm{V_{SHE}}$, -0.60 $\mathrm{V_{SHE}}$, -0.71 $\mathrm{V_{SHE}}$, respectively. To align our model with these observations, we adjust the pseudopotential parameter, v_{cc}^{0}, while keeping other parameters the same. The calibrated parameters for another two crystal Au and Ag facets are $v_{\mathrm{cc}}^{0,\mathrm{Au(111)}} = -0.572$ eV, $v_{\mathrm{cc}}^{0,\mathrm{Au(100)}} = -0.564$ eV and $v_{\mathrm{cc}}^{0,\mathrm{Ag(100)}} = -0.363$ eV, $v_{\mathrm{cc}}^{0,\mathrm{Ag(110)}} = -0.343$ eV, respectively. The more positive PZFC for the (111) facet correlates with its higher work function, which is captured in this model using a more negative v_{cc}^{0}. The observed discrepancies between model-based and experimental C_{dl} may arise from the orientation-dependent adsorption free energy of water[11] or ion partial desolvation at highly charged surfaces[8], which are not explicitly accounted for in the current model. To the best of our knowledge, these experimental C_{dl} data are systematically analyzed within a single modeling framework for the first time.

While the present DPFT framework successfully captures the C_{dl} behavior of noble metal electrodes, its current formulation is developed for metallic electrodes, where the electronic density of states (DOS) near the Fermi level is relatively smooth and leads to a monotonic dependence of electronic charge on electrode potential. In contrast, many carbon-based materials, such as graphene, exhibit a V-shaped DOS with a Dirac point, which can produce a non-monotonic charge response and a qualitatively different quantum capacitance behavior[12–14]. Capturing such effects would require incorporating an explicit description of the material-specific DOS into the electronic free-energy functional. While this aspect is not included in the current model, the DPFT framework could be extended in this direction, which may enable its application to carbonaceous and other non-metallic electrodes.

2. Effects of step density and height on the change in the PZFC

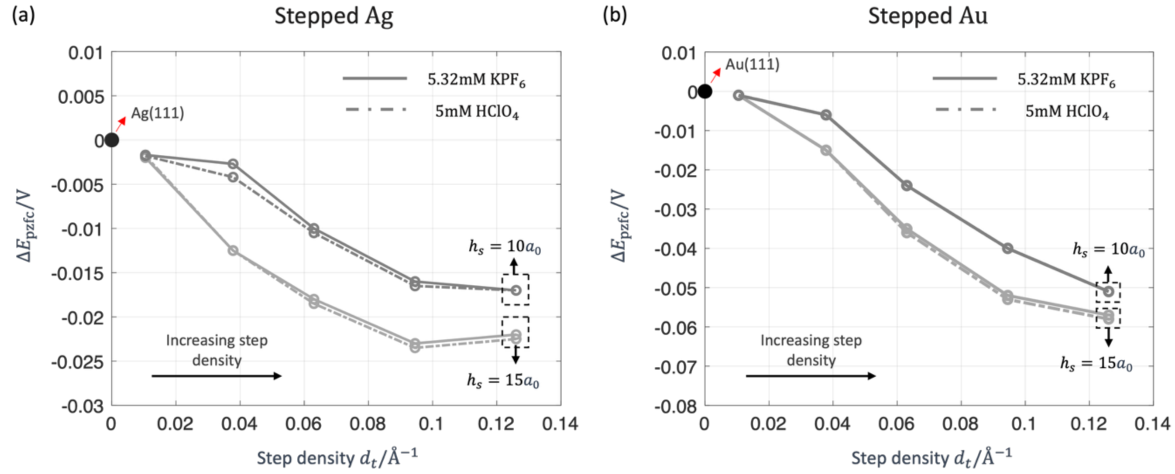

Figure S2. Step density and step height effects on the ΔE_{pzfc} of (a) stepped Ag and (b) stepped Au electrode in weakly adsorbing electrolytes. Model parameters are obtained at flat electrodes without any change.

3. Effects of step density and height on the electron spillover at PZFC

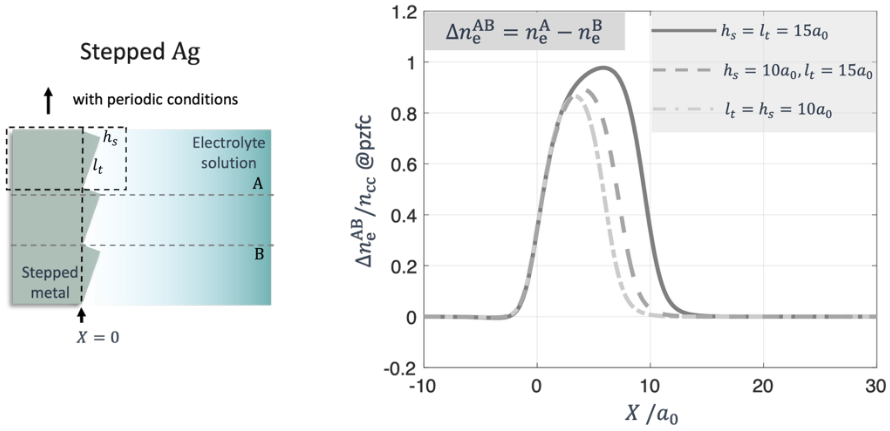

Figure S3. The difference of electron density between the position A and B (shown in the left figure) $\Delta n_e^{AB} = n_e^A - n_e^B$ at different terrace width l_t and step height h_s at the PZFC for stepped Ag in 5.32mM KPF_6 solution.

4. Step density dependence of the PZTC on Pt electrode

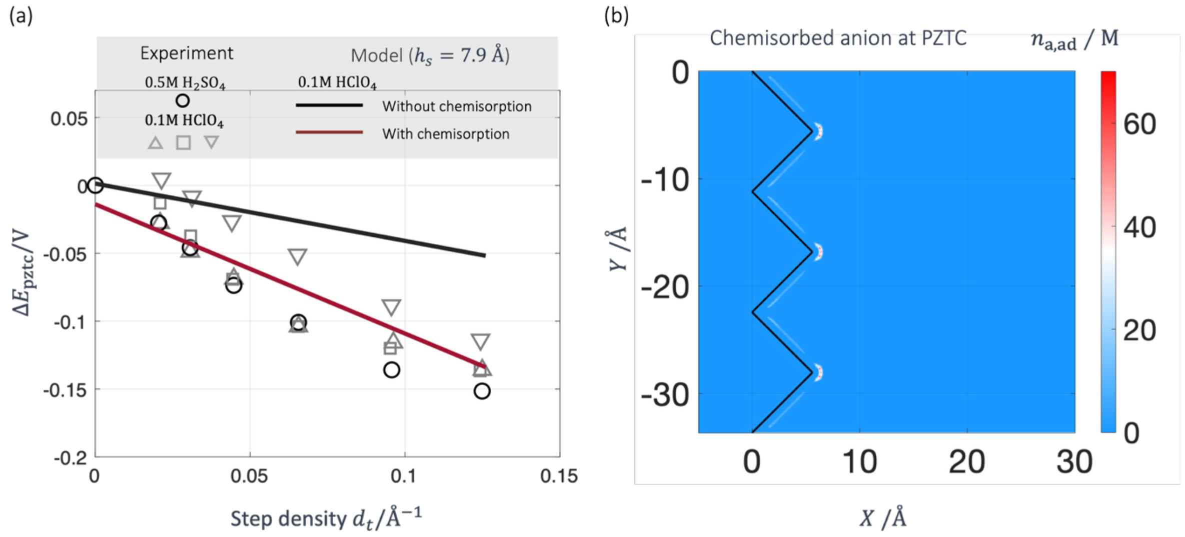

Figure S4. (a) Step density dependence of the PZTC referenced to the PZTC of flat single crystal electrodes for stepped Pt ($l_t = h_s = 15a_0 = 7.9$ Å) electrodes in 100mM $HClO_4$ solution for the case of without and with chemisorption. (b) the chemisorbed anion distribution at the PZTC. The model predictions obtained with model parameters listed in Table S1-S3 (solid line) are compared with experimental data (symbols) taken from ref.[15–17]. Model results are shown for a step height $h_s = 7.9$ Å.

5. Equivalence of surface tension and excess grand-canonical potential density

The surface tension γ is obtained by integrating the σ_{free} with respect to the electrode potential, up to a constant at the PZFC, as given in Eq.(S37). The excess grand-canonical potential density, G_{exe}, is independently calculated by spatial integration of grand-canonical potential in one dimension, as described in Eq.(S38). Figure S5 demonstrates excellent agreement between $\Delta\gamma_{\mathrm{st}}$ and ΔF_{int} for both $\mathrm{Au(110)}$ and $\mathrm{Ag(111)}$ electrodes, thereby confirming the thermodynamic consistency and numerical accuracy of the DPFT model. The small discrepancies are attributed to numerical processing techniques employed in the DPFT implementation to ensure stability and convergence, as discussed in the Supporting Information of ref.[18]. The surface tension profiles exhibit a parabolic shape with a maximum value at the PZFC in the absence of specific adsorption, in line with the electrocapillarity of EDLs[19].

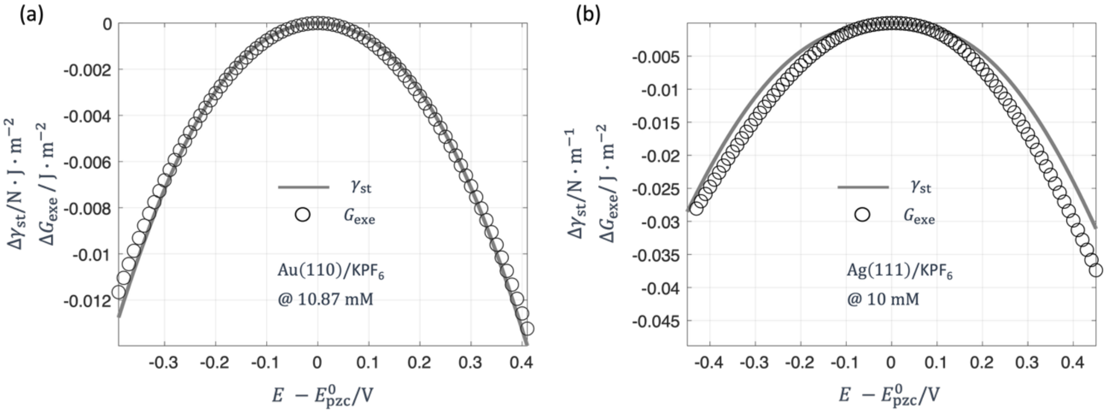

Figure S5. Comparison of two methods for calculating the surface tension. Relative surface tension (solid line) and relative excess grand-canonical potential density (circle) of solid metal-solution interfaces referred to the values at the PZFC for (a) $\mathrm{Au(110)}$@10.87 mM $\mathrm{KPF_6}$ and (b) $\mathrm{Ag(111)}$@10 mM $\mathrm{KPF_6}$ aqueous solution. The electrode potential is referenced to the PZFC.

6. Effect of step density on the surface charge density

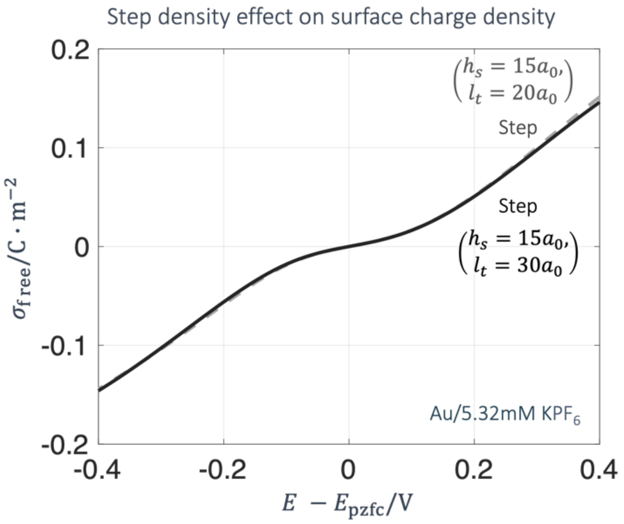

Figure S6. Effect of step density on surface charge density for stepped Ag in 5.32mM KPF_6 solution.

7. Ionic concentration effect on step bunching

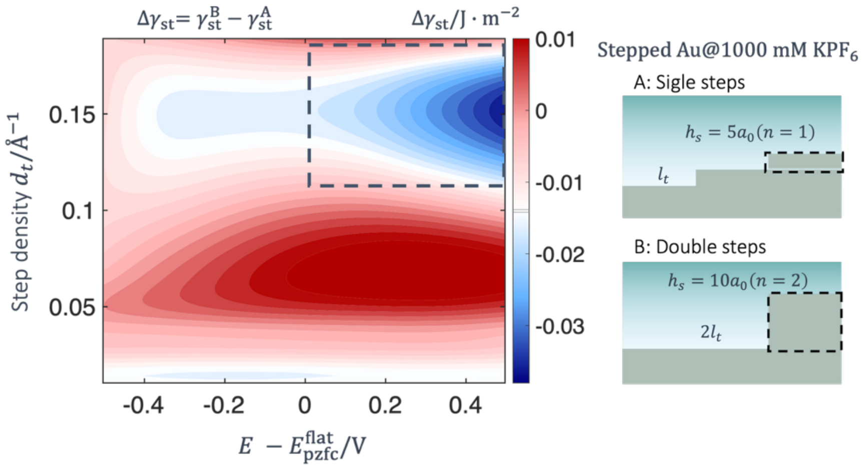

Figure S7. Phase map of the change in surface tension, $\Delta\gamma_{st} = \gamma_{st}^{B} - \gamma_{st}^{A}$, between double step (B) and single-step (A) configurations for stepped Au in $1000\mathrm{mM}\ KPF_6$ solution, as a function of step density d_t and electrode potential relative to the PZFC of the flat surface. Negative values indicate a thermodynamic driving force for step bunching. Insets schematically illustrate the single- and double-step geometries.

8. Theoretical and Computational Methods

The theoretical foundations and detailed derivation of the DPFT have been presented in our previous works[18,20] and are briefly summarized here for completeness. The volumetric grand potential g of the electric double layer (EDL) is written as,

$$g = f_{\mathrm{qm}}[n_{\mathrm{e}}, \nabla n_{\mathrm{e}}] + f_{\mathrm{c}}[\phi, \nabla\phi, \{n_{\mathrm{i}}\}] + f_{\mathrm{int}}[\phi, \nabla\phi, \{n_{\mathrm{i}}\}] - \left[n_{\mathrm{e}}\tilde{\mu}_{\mathrm{e}} + \sum_{\mathrm{i=a,c,s}} n_{\mathrm{i}}\tilde{\mu}_{\mathrm{i}}\right], \tag{S1}$$

where f_{qm}, f_{c} and f_{int} represent the three contributions to the Helmholtz free energy, namely, the quantum part for the electron gas, the classical part for the electrolyte solution, and the short-range interactions between the electron gas and the electrolyte solution, respectively. The last term ensures modelling the EDL as an open system in contact with both an electron reservoir and an electrolyte solution reservoir. $\tilde{\mu}_{\mathrm{e}}$ and $\tilde{\mu}_{\mathrm{i}}(\mathrm{i} = \mathrm{c, a, s})$ represent the electrochemical potentials of electrons and solution species, respectively. f_{qm} is written as,

$$\begin{aligned} f_{\mathrm{qm}} &= t_{\mathrm{ni}}[n_e, \nabla n_e, \ldots] + u_{\mathrm{X}}[n_e, \nabla n_e, \ldots] + u_{\mathrm{C}}[n_e, \nabla n_e, \ldots] + p_{\mathrm{cc}}[n_e] \\ &= e_{\mathrm{au}} a_0^{-3}[t_{\mathrm{TF}}(F_s(s) + \theta_{\mathrm{T}} s^2) + u_{\mathrm{X}}^0(1 + \theta_{\mathrm{X}} s^2) + (u_{\mathrm{C}}^0 + \theta_{\mathrm{C}} n_{\mathrm{e}} a_0^3 t^2) + v_{\mathrm{cc}} n_{\mathrm{e}}], \end{aligned} \tag{S2}$$

where $e_{\mathrm{au}} = e_0^2/(4\pi\epsilon_0 a_0)$ is the atomic energy with a_0 being the Bohr radius, e_0 the unit of electron charge, and ϵ_0 the vacuum permittivity. The kinetic energy of electrons is described by the semilocal approximation functionals[21–23]. $t_{\mathrm{TF}} = \frac{3}{10}(3\pi^2)^{\frac{2}{3}}(n_{\mathrm{e}} a_0^3)^{\frac{5}{3}}$ is the Thomas-Fermi volumetric kinetic energy density, and $(F_s(s) + \theta_{\mathrm{T}} s^2)$ is the correction for inhomogeneity. $F_s(s)$ is the so-called Pauli enhancement factor, $s = |\nabla n_{\mathrm{e}}| / \left(2(3\pi^2)^{\frac{1}{3}}(n_{\mathrm{e}})^{\frac{4}{3}}\right)$ is the reduced gradient term and θ_{T} is a gradient coefficient tuning the contribution of the gradient term. Here we take $\theta_{\mathrm{T}} = \frac{5}{3}$, a value commonly used in describing the metal[21–23]. In this letter, we employ the Thomas-Fermi-von Weiszäcker (TFvW) functionals, which is widely used for modeling transition metals[23]. This modifies the enhancement term in Eq.(S2), replaced $F_s(s)$ with 1. The exchange-correlation energy is described by the PBE functional with the exchange energy of a uniform electron gas, $u_{\mathrm{X}}^0 = -\frac{3}{4}\left(\frac{3}{\pi}\right)^{\frac{1}{3}}(n_{\mathrm{e}} a_0^3)^{\frac{4}{3}}$, $\theta_{\mathrm{X}} = 0.1235$ is the variable tuning the contribution of the gradient term in the exchange energy. Similarly, u_{C}^0 is the correlation energy of a uniform electron gas, for which we use the interpolation relation of Perdew et al.,[21]

$$u_{\mathrm{C}}^0 = -2\alpha_1 n_{\mathrm{e}} a_0^3(1 + \alpha_2 r_s) \ln\left(1 + \frac{1}{\xi}\right), \tag{S3}$$

$$r_s = \left(\frac{3}{4\pi n_\mathrm{e} a_0^3}\right)^{\frac{1}{3}},$$

$$\xi = 2\alpha_1 \left(\alpha_3 r_s^{\frac{1}{2}} + \alpha_4 r_s + \alpha_5 r_s^{\frac{3}{2}} + \alpha_6 r_s^2\right),$$

where $\alpha_1 = 0.0310907, \alpha_2 = 0.21370, \alpha_3 = 7.5957, \alpha_4 = 3.5876, \alpha_5 = 1.6382, \alpha_6 = 0.49294$. $\theta_\mathrm{C} = 0.0667$ is a gradient coefficient in the correlation energy and $t = a_0^4 |\nabla n_\mathrm{e}| / \left(4\left(\frac{3}{\pi}\right)^{\frac{1}{6}} (n_\mathrm{e} a_0^3)^{\frac{7}{6}}\right)$ is another reduced density gradient in terms of dimensional n_e and coordinates. v_cc is the pseudopotential of metal cationic cores effectively describing the influence of metal cationic cores on valence electrons in addition to the mean-field electrostatic force that is included elsewhere. The decay of v_cc from its bulk value v_cc^0 within the metal to zero outside the metal surface is modeled using an error function,

$$v_\mathrm{cc} = \frac{v_\mathrm{cc}^0}{2}[1 - \mathrm{erf}(x)]. \tag{S4}$$

It should be noted that in this work v_cc^0 is calibrated to reproduce the experimentally measured potential of zero charge. f_c describes the classical interactions between charged particles, which is a functional of the density of charged particles n_i and electric potential ϕ and its gradient $\nabla\phi$, written as,

$$f_\mathrm{c} = \left[(n_\mathrm{cc} - n_\mathrm{e})e_0\phi - \frac{1}{2}\epsilon_\mathrm{op}(\nabla\phi)^2\right]$$
$$+ \left[(n_\mathrm{c} - n_\mathrm{a})e_0\phi - n_\mathrm{s}\beta^{-1}\ln\frac{\sinh(\beta p_\mathrm{s}|\nabla\phi|)}{\beta p_\mathrm{s}|\nabla\phi|}\right] \tag{S5}$$
$$+ \sum_{\mathrm{i=a,c,s}} \beta^{-1} n_\mathrm{i}(\ln(n_\mathrm{i}\Lambda_\mathrm{i}^3) - 1) + \Phi_\mathrm{ex}(\{n_\mathrm{i}\}),$$

where n_cc is the number density of metal atomic-core charges, as only the valence electrons are explicitly considered in n_e. We can obtain the dimensionless $\bar{n}_\mathrm{cc}^\mathrm{Au} = 4N_\mathrm{Au}^\mathrm{ve}\left(\frac{a_0}{a_\mathrm{Au}}\right)^3 = 0.0959$ with $N_\mathrm{Au}^\mathrm{ve} = 11$ representing the number of valence electrons of an Au atom, and $a_\mathrm{Au} = 4.08$ Å is the lattice constant of the cubic closed-packed cell of Au, which contains four gold atoms. Similarly, we have $\bar{n}_\mathrm{cc}^\mathrm{Ag} = 4N_\mathrm{Ag}^\mathrm{ve}\left(\frac{a_0}{a_\mathrm{Ag}}\right)^3 = 0.0952$ with $N_\mathrm{Ag}^\mathrm{ve} = 11$ and $a_\mathrm{Ag} = 4.09$ Å. The first term on the right-hand side of Eq.(S5) represents the Hartree energy of electrons and cationic cores of the electrode, and the self-energy of the electric field with ϵ_op being the optical permittivity taken $9.8\epsilon_0$ for Au and $3\epsilon_0$ for Ag[24,25]. The second term is the potential energies of charged particles in solution, with $n_\mathrm{i}(\mathrm{i = a, c, s})$ the number density of

anion, cation and solvent, $\beta = (k_B T)^{-1}$ the inverse thermal energy, and p_s the solvent dipole moment. The third term represents the Gibbs free energy of an ideal-gas reference system, with Λ_i denoting the thermal wavelength of species i. The fourth term, $\Phi_{ex}(\{n_i\})$, accounts for excess Gibbs free energy due to finite-size effects. In this work, we adopt the Bikerman theory, a lattice gas approach that incorporates entropy of mixing in electrolyte solution[26–28]. The Bikerman theory assumes all charged particles occupy the same volume Λ_B, giving a maximum number density of $n_{max} = (\Lambda_B)^{-3}$. f_{int} accounts for the specific short-range interactions between solution particles and metal electron, which is written as,

$$f_{int} = \sum_{i=a,c,s} n_i w_i. \tag{S6}$$

Following our recent work[8], we employ the repulsive part of the Morse potential to prevent ions and solvent molecules from penetrating into the metal phase. This interaction is written as,

$$w_i(\vec{r}) = D_{mi} \cdot \exp\left(-2\beta_i(d(\vec{r}) - d_{mi})\right), \tag{S7}$$

where D_{mi} is the well depth, β_i is a coefficient controlling the well width, $d(\vec{r})$ is the distance from $\vec{r}$ to the metal surface edge, and d_{mi} denotes the equilibrium distance between the particle i and the metal surface. When $\vec{r}$ is within the metal, $d(\vec{r})$ is negative and $w_i(\vec{r})$ becomes very positive, meaning that solution particles have a negligible probability there. The parameters in Eq.(S7) can be determined from the Kohn-Sham DFT calculations[29]. For example, the DFT-calculated binding energy of water molecule on metal surface is approximately 0.25 eV, namely, $D_{ms} = 0.25$ eV, and the typical equilibrium distance between the water molecules and the metal surface is about 1.0 Å, corresponding to $d_{ms} = 1.89\ a_0$. This value appears smaller than the DFT-calculated bond length of 1.25 Å because the jellium edge is located approximately half a lattice constant beyond the outer surface of metal atoms[30–33].

Variational analysis

Variational analysis of g in terms of ϕ gives,

$$\frac{\partial g}{\partial \phi} - \nabla \cdot \left(\frac{\partial g}{\partial \nabla \phi}\right) = 0, \tag{S8}$$

leading to,

$$-\nabla[\epsilon_{eff} \nabla \phi] = e_0(n_{cc} - n_e) + (n_c - n_a)e_0, \tag{S9}$$

which is the Poisson equation with an effective dielectric function as in ref.[34–36]

$$\epsilon_{eff} = \epsilon_{op} + \frac{n_s p_s}{|\nabla \phi|} \mathcal{L}(\beta p_s |\nabla \phi|), \tag{S10}$$

with $\mathcal{L}(x) = \coth(x) - (x)^{-1}$ being the Langevin function.

Variational analysis of g in terms of particle number densities n_{i} should be divided into two cases. For the case of electrons, we obtain,

$$\nabla \cdot \left[\frac{\partial(t_{\mathrm{ni}} + u_{\mathrm{X}} + u_{\mathrm{C}})}{\partial \nabla n_{\mathrm{e}}}\right] = \frac{\partial(t_{\mathrm{ni}} + u_{\mathrm{X}} + u_{\mathrm{C}} + p_{\mathrm{cc}})}{\partial n_{\mathrm{e}}} - e_0\phi - \tilde{\mu}_{\mathrm{e}}. \tag{S11}$$

The electrochemical potential of electrons can be tuned by the electrode potential, E_{M},

$$\tilde{\mu}_{\mathrm{e}} = \mu_{\mathrm{e}} - e_0 E_{\mathrm{M}}, \tag{S12}$$

with $\mu_{\mathrm{e}} = \frac{\partial t_{\mathrm{TF}}}{\partial \bar{n}_{\mathrm{e}}} + \frac{\partial u_{\mathrm{X}}^0}{\partial \bar{n}_{\mathrm{e}}} + \frac{\partial u_{\mathrm{C}}^0}{\partial \bar{n}_{\mathrm{e}}} + v_{\mathrm{cc}}$ being the chemical potential of a homogenous electron gas. Eq.(S9) and (S11) constitute the basic set of differential equations controlling the EDL. Next, we need to derive expressions of n_{i} as functions of ϕ. Variational analysis of g in terms of charged particles in solution gives the electrochemical potential of ions and solvent,

$$\begin{aligned}
\tilde{\mu}_{\mathrm{a}} &= \beta^{-1}\ln(n_{\mathrm{a}}\Lambda_{\mathrm{a}}^3) - e_0\phi + w_{\mathrm{a}} + \mu_{\mathrm{a}}^{\mathrm{ex}}, \\
\tilde{\mu}_{\mathrm{c}} &= \beta^{-1}\ln(n_{\mathrm{c}}\Lambda_{\mathrm{c}}^3) + e_0\phi + w_{\mathrm{c}} + \mu_{\mathrm{c}}^{\mathrm{ex}}, \\
\tilde{\mu}_{\mathrm{s}} &= \beta^{-1}\ln(n_{\mathrm{s}}\Lambda_{\mathrm{s}}^3) - \beta^{-1}\ln\frac{\sinh(\beta p_{\mathrm{s}}|\nabla\phi|)}{\beta p_{\mathrm{s}}|\nabla\phi|} + w_{\mathrm{s}} + \mu_{\mathrm{s}}^{\mathrm{ex}},
\end{aligned} \tag{S13}$$

where $\mu_{\mathrm{i}}^{\mathrm{ex}}$ is the excess chemical potential given by,

$$\mu_{\mathrm{i}}^{\mathrm{ex}} = \frac{\delta \Phi_{\mathrm{ex}}}{\delta n_{\mathrm{i}}}. \tag{S14}$$

The Bikerman theory gives,

$$\mu_{\mathrm{i}}^{\mathrm{ex}} = \beta^{-1}\ln\left(\frac{1}{1 - \sum_{\mathrm{i=a,c,s}} n_{\mathrm{i}}\Lambda_{\mathrm{B}}^3}\right) \tag{S15}$$

A more advanced description is the fundamental measure theory (FMT)[26–28,37,38], which has been compared with the Bikerman theory in a previous work[39].

From Eq.(S13), n_i is given by,

$$\frac{n_\mathrm{i}\Lambda_\mathrm{i}^3}{1-\sum_{\mathrm{i=a,c,s}} n_\mathrm{i}\Lambda_\mathrm{B}^3} = \Theta_\mathrm{i}\exp(\beta\tilde{\mu}_\mathrm{i}), \tag{S16}$$

where thermodynamic factors are given by,

$$\Theta_\mathrm{a} = \exp(\beta e_0\phi - \beta w_\mathrm{a}),$$

$$\Theta_\mathrm{c} = \exp(-\beta e_0\phi - \beta w_\mathrm{c}), \tag{S17}$$

$$\Theta_\mathrm{s} = \exp\left(\ln\frac{\sinh(\beta p_\mathrm{s}|\nabla\phi|)}{\beta p_\mathrm{s}|\nabla\phi|} - \beta w_\mathrm{s}\right).$$

Eq.(S17) shall be valid also in the solution bulk where $\Theta_\mathrm{i} = 1$, and $\tilde{\mu}_\mathrm{i}$ is uniform in the electrolyte solution. Combining these two conditions, we have the following equality,

$$\frac{n_\mathrm{i}\Lambda_\mathrm{i}^3}{1-n_\mathrm{a}^\mathrm{b}\Lambda_\mathrm{B}^3-n_\mathrm{c}^\mathrm{b}\Lambda_\mathrm{B}^3-n_\mathrm{s}^\mathrm{b}\Lambda_\mathrm{B}^3} = \Theta_\mathrm{i}\frac{n_\mathrm{i}^\mathrm{b}\Lambda_\mathrm{i}^3}{1-n_\mathrm{a}^\mathrm{b}\Lambda_\mathrm{B}^3-n_\mathrm{c}^\mathrm{b}\Lambda_\mathrm{B}^3-n_\mathrm{s}^\mathrm{b}\Lambda_\mathrm{B}^3}. \tag{S18}$$

We have,

$$n_\mathrm{i} = n_\mathrm{max}\frac{\chi_\mathrm{i}\Theta_\mathrm{i}}{1+\chi_\mathrm{a}(\Theta_\mathrm{a}-1)+\chi_\mathrm{c}(\Theta_\mathrm{c}-1)+\chi_\mathrm{s}(\Theta_\mathrm{s}-1)}. \tag{S19}$$

with dimensionless bulk number densities $\chi_\mathrm{i} = n_\mathrm{i}^\mathrm{b}/n_\mathrm{max}$.

Eq.(S19) can be extended, in a phenomenological manner, to scenarios of unequal sizes,

$$n_\mathrm{i} = n_\mathrm{max}\frac{\chi_\mathrm{i}\Theta_\mathrm{i}}{\Omega}. \tag{S20}$$

where $\Omega = 1+\gamma_\mathrm{a}\chi_\mathrm{a}(\Theta_\mathrm{a}-1)+\gamma_\mathrm{c}\chi_\mathrm{c}(\Theta_\mathrm{c}-1)+\gamma_\mathrm{s}\chi_\mathrm{s}(\Theta_\mathrm{s}-1)$ is the normalization factor, and γ_i is the relative size of particles of the type i referenced to Λ_B.

Numerical implementation

In this section, we further process the governing equations in Eq.(S9) and (S11) to facilitate numerical computations. We define dimensionless variables, marked with overlines, as follows:

$$\bar{n}_{\mathrm{i}} = n_{\mathrm{i}}(a_0)^3, \bar{x} = \frac{x}{a_0}, \bar{\phi} = \frac{e_0\phi}{k_{\mathrm{B}}T}, \bar{p}_{\mathrm{s}} = \frac{p_{\mathrm{s}}}{e_0 a}, \bar{q}_{\mathrm{i}} = \frac{q_{\mathrm{i}}}{e_0}, \bar{\epsilon}_{\mathrm{op}} = \frac{\epsilon_{\mathrm{op}}}{\epsilon_0}$$

where ϵ_0 is the dielectric permittivity of vacuum. Then we rewrite Eq.(S9) in a nondimensional form,

$$-\bar{\nabla}\cdot\left(\bar{\epsilon}_{\mathrm{op}}\bar{\nabla}\bar{\phi} + \bar{n}_{\mathrm{s}}\bar{p}_{\mathrm{s}}\kappa\mathcal{L}\right) = \kappa\big((\bar{n}_{\mathrm{cc}} - \bar{n}_{\mathrm{e}}) + (\bar{n}_{\mathrm{c}} - \bar{n}_{a})\big), \tag{S21}$$

where $\kappa = \frac{e_0^2}{k_{\mathrm{B}}T\epsilon_0 a_0}$ is a number derived from fundamental constants.

We can rewrite Eq.(S11) in terms of the dimensionless electron density, $\bar{n}_{\mathrm{e}} = n_{\mathrm{e}}a_0^3$,

$$\bar{\nabla}\cdot\left[\frac{\partial(t_{\mathrm{ni}} + u_{\mathrm{X}} + u_{\mathrm{C}})}{\partial\bar{\nabla}\bar{n}_{\mathrm{e}}}\right] = \frac{\partial(t_{\mathrm{ni}} + u_{\mathrm{X}} + u_{\mathrm{C}} + p_{\mathrm{cc}})}{\partial\bar{n}_{\mathrm{e}}} - a_0^{-3}(e_0\phi + \tilde{\mu}_{\mathrm{e}}), \tag{S22}$$

where the terms are obtained as,

$$\frac{\partial(t_{\mathrm{ni}} + u_{\mathrm{X}} + u_{\mathrm{C}})}{\partial\bar{\nabla}\bar{n}_{\mathrm{e}}} = \frac{\partial(t_{\mathrm{ni}} + u_{\mathrm{X}} + u_{\mathrm{C}})}{\partial s^2}\frac{\partial s^2}{\partial\bar{\nabla}\bar{n}_{\mathrm{e}}}$$

$$= \frac{e_{\mathrm{au}}a_0^{-3}\left(\left(\frac{\partial F_s(s)}{\partial s^2} + \theta_{\mathrm{T}}\right)t_{\mathrm{TF}} + \theta_{\mathrm{XC}}u_{\mathrm{X}}^0\right)}{2(3\pi^2)^{\frac{2}{3}}(\bar{n}_{\mathrm{e}})^{\frac{8}{3}}}\bar{\nabla}\bar{n}_{\mathrm{e}}, \tag{S23}$$

with $\theta_{\mathrm{XC}} = \theta_{\mathrm{X}} - \frac{\pi^2}{3}\theta_{\mathrm{C}}$, and

$$\frac{\partial(t_{\mathrm{ni}} + u_{\mathrm{X}} + u_{\mathrm{C}} + p_{\mathrm{cc}})}{\partial\bar{n}_{\mathrm{e}}}$$

$$= e_{\mathrm{au}}a_0^{-3}[(F_s(s) + \theta_{\mathrm{T}}s^2)\frac{\partial t_{\mathrm{TF}}}{\partial\bar{n}_{\mathrm{e}}} + (1 + \theta_{\mathrm{XC}}s^2)\frac{\partial u_{\mathrm{X}}^0}{\partial\bar{n}_{\mathrm{e}}} + \frac{\partial u_{\mathrm{C}}^0}{\partial\bar{n}_{\mathrm{e}}} + \left(\left(\frac{\partial F_s(s)}{\partial s^2} + \theta_{\mathrm{T}}\right)t_{\mathrm{TF}} + \theta_{\mathrm{X}}'u_{\mathrm{X}}^0\right)\frac{\partial s^2}{\partial\bar{n}_{\mathrm{e}}} + v_{\mathrm{cc}}], \tag{S24}$$

with,

$$\frac{\partial t_{\mathrm{TF}}}{\partial \bar{n}_{\mathrm{e}}} = \frac{1}{2}(3\pi^2)^{\frac{2}{3}}(\bar{n}_{\mathrm{e}})^{\frac{2}{3}} \tag{S25}$$

$$\frac{\partial s^2}{\partial \bar{n}_{\mathrm{e}}} = -\frac{8}{3}\frac{(\bar{\nabla}\bar{n}_e)^2}{4(3\pi^2)^{\frac{2}{3}}(\bar{n}_{\mathrm{e}})^{\frac{11}{3}}} = \frac{-8}{3\bar{n}_{\mathrm{e}}}s^2 \tag{S26}$$

$$\frac{\partial u_{\mathrm{X}}^0}{\partial \bar{n}_{\mathrm{e}}} = -\left(\frac{3}{\pi}\right)^{\frac{1}{3}}(\bar{n}_{\mathrm{e}})^{\frac{1}{3}} \tag{S27}$$

$$\frac{\partial u_{\mathrm{C}}^0}{\partial \bar{n}_{\mathrm{e}}} = -2\alpha_1(1+\alpha_2 r_s)\ln\left(1+\frac{1}{\xi}\right) - 2\alpha_1\bar{n}_e\left(-\frac{1}{3}\left(\frac{3}{4\pi}\right)^{\frac{1}{3}}(\bar{n}_{\mathrm{e}})^{-\frac{4}{3}}\right)\cdot$$

$$\left(\alpha_2\ln\left(1+\frac{1}{\xi}\right) - \frac{(1+\alpha_2 r_s)}{\xi(1+\xi)}\alpha_1\left(\alpha_3 r_s^{-\frac{1}{2}} + 2\alpha_4 + 3\alpha_5 r_s^{\frac{1}{2}} + 4\alpha_6 r_s\right)\right) \tag{S28}$$

$$= -2\alpha_1(1+\alpha_2 r_s)\ln\left(1+\frac{1}{\xi}\right)$$

$$+\frac{2\alpha_1 r_s}{3}\left(\alpha_2\ln\left(1+\frac{1}{\xi}\right) - \frac{\alpha_1(1+\alpha_2 r_s)}{\xi(1+\xi)}\left(\alpha_3 r_s^{-\frac{1}{2}} + 2\alpha_4 + 3\alpha_5 r_s^{\frac{1}{2}} + 4\alpha_6 r_s\right)\right).$$

We expand the term on the right most side of Eq.(S23),

$$\bar{\nabla}\cdot\left[\frac{\left(\left(\frac{\partial F_s(s)}{\partial s^2}+\theta_{\mathrm{T}}\right)t_{\mathrm{TF}}+\theta_{\mathrm{XC}}u_{\mathrm{X}}^0\right)}{(\bar{n}_{\mathrm{e}})^{\frac{8}{3}}}\bar{\nabla}\bar{n}_{\mathrm{e}}\right] = \frac{\left(\left(\frac{\partial F_s(s)}{\partial s^2}+\theta_{\mathrm{T}}\right)t_{\mathrm{TF}}+\theta_{\mathrm{XC}}u_{\mathrm{X}}^0\right)}{(\bar{n}_{\mathrm{e}})^{\frac{8}{3}}}\bar{\nabla}^2\bar{n}_{\mathrm{e}} \tag{S29}$$

$$-\bar{\nabla}\cdot\left[\frac{\left(\left(\frac{\partial F_s(s)}{\partial s^2}+\theta_{\mathrm{T}}\right)t_{\mathrm{TF}}+\theta_{\mathrm{XC}}u_{\mathrm{X}}^0\right)}{(\bar{n}_{\mathrm{e}})^{\frac{8}{3}}}\right]\bar{\nabla}\bar{n}_{\mathrm{e}}$$

$$= \frac{\left(\left(\frac{\partial F_s(s)}{\partial s^2} + \theta_{\mathrm{T}}\right) t_{\mathrm{TF}} + \theta_{\mathrm{XC}} u_{\mathrm{X}}^0\right)}{(\bar{n}_{\mathrm{e}})^{\frac{8}{3}}} \bar{\nabla}^2 \bar{n}_{\mathrm{e}}$$

$$+ \frac{\left(\frac{\partial F_s(s)}{\partial s^2 \partial \bar{n}_{\mathrm{e}}} t_{\mathrm{TF}} + \left(\frac{\partial F_s(s)}{\partial s^2} + \theta_{\mathrm{T}}\right) \frac{\partial t_{\mathrm{TF}}}{\partial \bar{n}_{\mathrm{e}}} + \theta_{\mathrm{XC}} \frac{\partial u_{\mathrm{X}}^0}{\partial \bar{n}_{\mathrm{e}}}\right)}{(\bar{n}_{\mathrm{e}})^{\frac{8}{3}}} (\bar{\nabla} \bar{n}_{\mathrm{e}})^2$$

$$- \frac{8}{3} \frac{\left(\left(\frac{\partial F_s(s)}{\partial s^2} + \theta_{\mathrm{T}}\right) t_{\mathrm{TF}} + \theta_{\mathrm{XC}} u_{\mathrm{X}}^0\right)}{(\bar{n}_{\mathrm{e}})^{\frac{11}{3}}} (\bar{\nabla} \bar{n}_{\mathrm{e}})^2.$$

Combining Eq.(S22), (S23) and (S24), we get,

$$\bar{\nabla}^2 \bar{n}_{\mathrm{e}} = \frac{2(3\pi^2)^{\frac{2}{3}} (\bar{n}_{\mathrm{e}})^{\frac{8}{3}}}{\left(\frac{\partial F_s(s)}{\partial s^2} + \theta_{\mathrm{T}}\right) t_{\mathrm{TF}} + \theta_{\mathrm{XC}} u_{\mathrm{X}}^0} [(F_s(s) + \theta_{\mathrm{T}} s^2) \frac{\partial t_{\mathrm{TF}}}{\partial \bar{n}_{\mathrm{e}}} + (1 + \theta_{\mathrm{XC}} s^2) \frac{\partial u_{\mathrm{X}}^0}{\partial \bar{n}_{\mathrm{e}}} + \frac{\partial u_{\mathrm{C}}^0}{\partial \bar{n}_{\mathrm{e}}}$$
$$+ \left(\left(\frac{\partial F_s(s)}{\partial s^2} + \theta_{\mathrm{T}}\right) t_{\mathrm{TF}} + \theta_{\mathrm{XC}} u_{\mathrm{X}}^0\right) \frac{\partial s^2}{\partial \bar{n}_{\mathrm{e}}} + v_{\mathrm{cc}} - \frac{(e_0 \phi + \tilde{\mu}_{\mathrm{e}})}{e_{\mathrm{au}}}] \qquad \text{(S30)}$$

$$+ \frac{8}{3} (\bar{n}_{\mathrm{e}})^{-1} (\bar{\nabla} \bar{n}_{\mathrm{e}})^2 - \frac{\frac{\partial F_s(s)}{\partial s^2 \partial \bar{n}_{\mathrm{e}}} t_{\mathrm{TF}} + \left(\frac{\partial F_s(s)}{\partial s^2} + \theta_{\mathrm{T}}\right) \frac{\partial t_{\mathrm{TF}}}{\partial \bar{n}_{\mathrm{e}}} + \theta_{\mathrm{XC}} \frac{\partial u_{\mathrm{X}}^0}{\partial \bar{n}_{\mathrm{e}}}}{\left(\frac{\partial F_s(s)}{\partial s^2} + \theta_{\mathrm{T}}\right) t_{\mathrm{TF}} + \theta_{\mathrm{XC}} u_{\mathrm{X}}^0} (\bar{\nabla} \bar{n}_{\mathrm{e}})^2.$$

Substituting Eq.(S26) into Eq.(S30), and using $s = |\bar{\nabla} \bar{n}_{\mathrm{e}}| / \left(2(3\pi^2)^{\frac{1}{3}} (\bar{n}_{\mathrm{e}})^{\frac{4}{3}}\right)$, we get,

$$\bar{\nabla}^2 \bar{n}_{\mathrm{e}} = \frac{2(3\pi^2)^{\frac{2}{3}} (\bar{n}_{\mathrm{e}})^{\frac{8}{3}}}{\left(\frac{\partial F_s(s)}{\partial s^2} + \theta_{\mathrm{T}}\right) t_{\mathrm{TF}} + \theta_{\mathrm{XC}} u_{\mathrm{X}}^0} \left(F_s(s) \frac{\partial t_{\mathrm{TF}}}{\partial \bar{n}_{\mathrm{e}}} + \frac{\partial u_{\mathrm{X}}^0}{\partial \bar{n}_{\mathrm{e}}} + \frac{\partial u_{\mathrm{C}}^0}{\partial \bar{n}_{\mathrm{e}}} + v_{\mathrm{cc}} - \frac{(e_0 \phi + \tilde{\mu}_{\mathrm{e}})}{e_{\mathrm{au}}}\right)$$
$$\text{(S31)}$$
$$+ \left(\frac{4}{3} (\bar{n}_{\mathrm{e}})^{-1} - \frac{2 \frac{\partial F_s(s)}{\partial s^2 \partial \bar{n}_{\mathrm{e}}} t_{\mathrm{TF}} + \left(2 \frac{\partial F_s(s)}{\partial s^2} + \theta_{\mathrm{T}}\right) \frac{\partial t_{\mathrm{TF}}}{\partial \bar{n}_{\mathrm{e}}} + \theta_{\mathrm{XC}} \frac{\partial u_{\mathrm{X}}^0}{\partial \bar{n}_{\mathrm{e}}}}{2\left(\left(\frac{\partial F_s(s)}{\partial s^2} + \theta_{\mathrm{T}}\right) t_{\mathrm{TF}} + \theta_{\mathrm{XC}} u_{\mathrm{X}}^0\right)}\right) (\bar{\nabla} \bar{n}_{\mathrm{e}})^2.$$

Since, $\frac{\partial t_{\mathrm{TF}}}{\partial \bar{n}_{\mathrm{e}}} = \frac{5 t_{\mathrm{TF}}}{3 \bar{n}_{\mathrm{e}}}, \frac{\partial u_{\mathrm{X}}^0}{\partial \bar{n}_{\mathrm{e}}} = \frac{4 u_{\mathrm{X}}^0}{3 \bar{n}_{\mathrm{e}}}$, we obtain,

$$\frac{4}{3}(\bar{n}_{\mathrm{e}})^{-1}-\frac{2\frac{\partial F_s(s)}{\partial s^2\partial\bar{n}_{\mathrm{e}}}t_{\mathrm{TF}}+\left(2\frac{\partial F_s(s)}{\partial s^2}+\theta_{\mathrm{T}}\right)\frac{\partial t_{\mathrm{TF}}}{\partial\bar{n}_{\mathrm{e}}}+\theta_{\mathrm{XC}}\frac{\partial u_{\mathrm{X}}^0}{\partial\bar{n}_{\mathrm{e}}}}{2\left(\left(\frac{\partial F_s(s)}{\partial s^2}+\theta_{\mathrm{T}}\right)t_{\mathrm{TF}}+\theta_{\mathrm{XC}}u_{\mathrm{X}}^0\right)}$$
$$=\frac{\left(\theta_{\mathrm{T}}t_{\mathrm{TF}}+\frac{4}{3}\theta_{\mathrm{XC}}u_{\mathrm{X}}^0\right)-2\bar{n}_{\mathrm{e}}\frac{\partial F_s(s)}{\partial s^2\partial\bar{n}_{\mathrm{e}}}t_{\mathrm{TF}}-\frac{2}{3}\frac{\partial F_s(s)}{\partial s^2}t_{\mathrm{TF}}}{2\bar{n}_{\mathrm{e}}\left(\left(\frac{\partial F_s(s)}{\partial s^2}+\theta_{\mathrm{T}}\right)t_{\mathrm{TF}}+\theta_{\mathrm{XC}}u_{\mathrm{X}}^0\right)}. \tag{S32}$$

In the end, we reformulate Eq.(S31) as,

$$\bar{\nabla}^2\bar{n}_{\mathrm{e}}=\frac{20}{3}\bar{n}_{\mathrm{e}}\frac{\omega}{\left(\frac{\partial F_s(s)}{\partial s^2}+\theta_{\mathrm{T}}\right)\omega-\theta_{\mathrm{XC}}}\left(F_s(s)\frac{\partial t_{\mathrm{TF}}}{\partial\bar{n}_{\mathrm{e}}}+\frac{\partial u_{\mathrm{X}}^0}{\partial\bar{n}_{\mathrm{e}}}+\frac{\partial u_{\mathrm{C}}^0}{\partial\bar{n}_{\mathrm{e}}}+v_{\mathrm{cc}}-\frac{(e_0\phi+\tilde{\mu}_{\mathrm{e}})}{e_{\mathrm{au}}}\right)$$
$$+\frac{\theta_{\mathrm{T}}\omega-\frac{4}{3}\theta_{\mathrm{XC}}-2\bar{n}_{\mathrm{e}}\frac{\partial F_s(s)}{\partial s^2\partial\bar{n}_{\mathrm{e}}}\omega-\frac{2}{3}\frac{\partial F_s(s)}{\partial s^2}\omega}{2\bar{n}_{\mathrm{e}}\left(\left(\frac{\partial F_s(s)}{\partial s^2}+\theta_{\mathrm{T}}\right)\omega-\theta_{\mathrm{XC}}\right)}(\bar{\nabla}\bar{n}_{\mathrm{e}})^2. \tag{S33}$$

with $\omega=\frac{2}{5}\pi^{\frac{5}{3}}3^{\frac{1}{3}}(\bar{n}_{\mathrm{e}})^{\frac{1}{3}}$, which is the general form of the controlling equation for the electron density.

Calculation of thermodynamic properties

Within this framework, the C_{dl} is calculated by differentiating the surface free charge density σ_{free} with respect to electrode potential E_{M},

$$C_{\mathrm{dl}}=\frac{\partial\sigma_{\mathrm{free}}}{\partial E_{\mathrm{M}}}. \tag{S34}$$

$\tilde{\mu}_{\mathrm{e}}$ is related to the E_{M}, referenced to the standard hydrogen electrode (SHE) scale[40,41], as follows,

$$-\tilde{\mu}_{\mathrm{e}}=e_0(E_{\mathrm{M}}+4.44\ \mathrm{V})-e_0\chi_{\mathrm{s}}^{\mathrm{v}}, \tag{S35}$$

where $\chi_{\mathrm{s}}^{\mathrm{v}}$ is the surface potential at the solution-vacuum interface[42]. By applying this relationship, we can rewrite Eq.(S34),

$$C_{\mathrm{dl}}=-e_0\frac{\partial\sigma_{\mathrm{free}}}{\partial\tilde{\mu}_{\mathrm{e}}}=\frac{e_0^2}{a_0^2}\frac{\partial}{\partial\tilde{\mu}_{\mathrm{e}}}\int d\bar{x}(\bar{n}_{\mathrm{c}}-\bar{n}_{\mathrm{a}}), \tag{S36}$$

with $\sigma_{\mathrm{free}}=-\frac{e_0}{a_0^2}\int d\bar{x}(\bar{n}_{\mathrm{c}}-\bar{n}_{\mathrm{a}})$, where $\bar{n}_{\mathrm{c}}$ and $\bar{n}_{\mathrm{a}}$ represent the dimensionless number densities of cation and anion, respectively.

The surface tension γ_{st} can be obtained using the Lippmann equation, which is, up to a constant, the integral of σ_{free} over the electrode potential[19]. In this work, we examine the relative surface tension, $\Delta\gamma_{\mathrm{st}}$, referenced to their values at the PZC,

$$\Delta\gamma_{\mathrm{st}} = -\int_{E_{\mathrm{pzc}}}^{E_{\mathrm{M}}} \sigma_{\mathrm{free}} dE. \tag{S37}$$

The excess grand-canonical potential density, g_{exe}, is derived from the integration of the grand-canonical potential of interfaces over space as defined in Eq.(S1), written as,

$$\begin{aligned} \Delta G_{\mathrm{exe}} &= G_{\mathrm{exe}}(E_{\mathrm{M}}) - G_{\mathrm{exe}}(E_{\mathrm{pzc}}), \\ \Delta G_{\mathrm{exe}}(E_{\mathrm{M}}) &= \int_0^L g(E_{\mathrm{M}}) dx - \left(\int_0^{L_{\mathrm{M}}} g_{\mathrm{m}} dx + \int_{L_{\mathrm{M}}}^L g_{\mathrm{sol}} dx\right), \end{aligned} \tag{S38}$$

where the last two terms in the bracket are the volumetric grand-canonical potential density of the bulk metal and the bulk solution, respectively.

Table S1. Basic model parameters

Category	Symbol	Item	Value	Note
General constants	R	Ideal gas constant	$8.314\ \mathrm{JK^{-1}mol^{-1}}$	
	k_B	Boltzmann constant	$1.38 \cdot 10^{-23}$ J/K	
	T	Temperature	298 K	
	e_0	Elementary charge	1.6×10^{-19}C	
	e_au	Energy constant from arb. units to SI	27.2 eV	
	N_A	Avogadro's number	6.02×10^{23}/mol	
	ϵ_0	Vacuum permittivity	8.85×10^{-12} F/m	
	a_0	Bohr radius	5.29×10^{-11} m	
	n_ref	Reference number density	$(a_0)^{-3}$	
	κ	Dimensionless constant	$e_0^2/(k_\mathrm{B} T \epsilon_0 a_0)$	

Category	Symbol	Item	Value	Note
Electrolyte	v	Bulk volume fraction of solvated ions	$2{a_0}^3 n_0^\mathrm{b}$	
	$\bar{n}_\mathrm{s,total}^{\mathrm{H_2O}}$	Dimensionless total water number density	$5.5 \times 10^4 N_\mathrm{A}(a_0)^3$	Ref[43]
	R_sol	Diameter of solvent molecule	$1 \times 10^{10} a_0 \left(1/\bar{n}_\mathrm{s,total}\right)^{1/3}$ Å	
	$r_{\mathrm{H^+}}^{\mathrm{H_2O}}$	Radius of solvated H^+ in H_2O	4 Å	Estimated
	$r_{\mathrm{ClO_4^-}}^{\mathrm{H_2O}}$	Radius of solvated PF_6^- in H_2O	3.25 Å	Estimated
	$r_{\mathrm{K^+}}^{\mathrm{H_2O}}$	Radius of solvated K^+ in H_2O	3.5 Å	Estimated
	$r_{\mathrm{PF_6^-}}^{\mathrm{H_2O}}$	Radius of solvated PF_6^- in H_2O	3 Å	Estimated
	$\gamma_{\mathrm{c/s}}$	Relative size of solvated cations	$(2r_\mathrm{c}/R_\mathrm{s})^3$	Ref[44]
	$\gamma_{\mathrm{a/s}}$	Relative size of solvated anions	$(2r_\mathrm{a}/R_\mathrm{s})^3$	Ref[44]
	$\epsilon_\mathrm{s}^{\mathrm{H_2O}}$	Bulk relative permittivity of H_2O	78.5	
	$\chi_{\mathrm{H_2O}}^{\mathrm{v}}$	Surface potential of H_2O-vacuum interface	0.13 V	Ref[42]

Table S2. Parameters in the DPFT model

Category	Symbol	Item	Value	Note
Electrolyte	$\bar{\epsilon}_{\mathrm{op}}^{\mathrm{int,H_2O}}$	Optical permittivity of the Hg-aqueous solution interface	6	Ref[25]
	p_s	Solvent dipole moment	$\left[\frac{3*(\varepsilon_{\mathrm{r}}-\epsilon_{\mathrm{op}}^{\mathrm{int}})\epsilon_0 k_{\mathrm{B}}T}{n_{\mathrm{s,total}}N_{\mathrm{A}}}\right]$ D	Ref[44]
	χ_{v}	Volume fraction of vacancy in the bulk solution	0.05	
Metal	a_{Au}	the lattice constant of the cubic closed-packed cell of Au	4.08 Å	
	a_{Ag}	the lattice constant of the cubic closed-packed cell of Ag	4.09 Å	
	$\bar{n}_{\mathrm{cc}}^{0,\mathrm{Au}}$	Dimensionless metal valency electron density of Au	$\frac{4N_e^{\mathrm{Au}}}{\left(\frac{a_{\mathrm{Au}}}{a_0}\right)^3}=0.0959$	Considering eleven valency here, namely $N_e^{\mathrm{Au}}=11$
	$\bar{n}_{\mathrm{cc}}^{0,\mathrm{Ag}}$	Dimensionless metal valency electron density of Ag	$\frac{4N_e^{\mathrm{Ag}}}{\left(\frac{a_{\mathrm{Ag}}}{a_0}\right)^3}=0.0952$	Considering eleven valency here, namely $N_e^{\mathrm{Ag}}=11$
	$v_{\mathrm{cc}}^{0,\mathrm{Au(111)}}$	Dimensionless pseudopotential of Au(111) in the metal bulk	-0.572	Calibrated with experimental C_{dl} data
	$v_{\mathrm{cc}}^{0,\mathrm{Au(100)}}$	Dimensionless pseudopotential of Au(100) in the metal bulk	-0.564	
	$v_{\mathrm{cc}}^{0,\mathrm{Au(110)}}$	Dimensionless pseudopotential of Au(110) in the metal bulk	-0.549	
	$v_{\mathrm{cc}}^{0,\mathrm{Ag(111)}}$	Dimensionless pseudopotential of Ag(111) in the metal bulk	-0.394	
	$v_{\mathrm{cc}}^{0,\mathrm{Ag(100)}}$	Dimensionless pseudopotential of Ag(100) in the metal bulk	-0.363	
	$v_{\mathrm{cc}}^{0,\mathrm{Ag(110)}}$	Dimensionless pseudopotential of Ag(110) in the metal bulk	-0.343	
	$\bar{\epsilon}_{\mathrm{op}}^{\mathrm{Au}}$	Optical dielectric constant of metal Au	9.8	Ref[24]

	$\bar{\epsilon}_{\mathrm{op}}^{\mathrm{Ag}}$	Optical dielectric constant of metal Ag	3	
	θ_{T}	The gradient coefficients tuning the contribution of the semi-local term in kinetic energy of Thomas-Fermi von Weiszäcker (TFvW) functional	5/3	Ref[23]

Metal-electrolyte interactions	β_{l}	Coefficient in Morse potential	2	
	D_{ms}	Force constant of metal-solvent interactions	$0.25e_0/k_{\mathrm{B}}T$	Ref[45,46]
	$d_{\mathrm{mH_2O}}^{\mathrm{Au}}$	Metal Au-water equilibrium distance	1.0 Å	
	$d_{\mathrm{mH_2O}}^{\mathrm{Ag}}$	Metal Ag-water equilibrium distance	1.0 Å	
	$D_{\mathrm{ma(c)}}$	Force constant of metal-ion interactions	$D_{\mathrm{ms}}/6$	Calibrated with experimental C_{dl} data
	$d_{\mathrm{mK^+}}^{\mathrm{Au}}$	Metal Au-K^+ equilibrium distance	1.8 Å	
	$d_{\mathrm{mPF_6^-}}^{\mathrm{Au}}$	Metal Au-PF_6^- equilibrium distance	1.8 Å	
	$d_{\mathrm{mH^+}}^{\mathrm{Au}}$	Metal Au-H^+ equilibrium distance	2.8 Å	
	$d_{\mathrm{mClO_4^-}}^{\mathrm{Au}}$	Metal Au-ClO_4^- equilibrium distance	2.8 Å	
	$d_{\mathrm{mK^+}}^{\mathrm{Ag}}$	Metal Ag-K^+ equilibrium distance	4.8 Å	
	$d_{\mathrm{mPF_6^-}}^{\mathrm{Ag}}$	Metal Ag-PF_6^- equilibrium distance	1.8 Å	

Table S3. Parameters in the DPFT_chem model[47,48]

Symbol	Meaning	Value
D_{ca}^0	Intercept of the binding strength relation in Eq.(19) of ref[47] at $\tilde{\mu}_e = 0$ eV.	-1.08 eV
α_{ca}	Linear coefficient of the binding strength relation in Eq.(19) of ref[47]	-0.35
β_{ca}	Coefficient in the Morse potential of chemisorbing anions in Eq.(18) of ref[47]	$0.8/a_0$
b_{ca}	Location of the minimum of the Morse potential of chemisorbing anions in Eq.(18) of ref[47]	2.8 Å
Δ^0	Strength of electronic interactions between the metal and chemisorbing anions	2 eV
κ_Δ	Coefficient determining the exponential decay of electronic interactions	$1/a_0$

d_{cut}	Cutoff distance of the exponential decays	$5a_0$
ϵ_{ca}^0	Reference energy of the valence orbital of chemisorbing anions	-3.2 eV
Δ_ϵ	Overall change of the valence orbital of chemisorbing anions	3 eV
κ_ϵ	Coefficient determining the change rate of the valence orbital of chemisorbing anion	$1/a_0$

Reference

(1) Hamelin, A.; Stoicoviciu, L. Study of Gold Low Index Faces in KPF6 solutionsPART I. EXPERIMENTAL BEHAVIOUR AND DETERMINATION OF THJ3 POINTS OF ZERO CHARGE. *J. Electroanal. Chem. Interfacial Electrochem.* **1987**, *234* (1), 93–105. https://doi.org/10.1016/0022-0728(87)80164-X.

(2) Samec, Z.; Doblhofer, K. Mechanism of Peroxodisulfate Reduction at a Polycrystalline Gold Electrode. *J. Electroanal. Chem.* **1994**, *367* (1), 141–147. https://doi.org/10.1016/0022-0728(93)03041-M.

(3) Hamelin, A.; Stoicoviciu, L.; Silva, F. The Temperature Dependence of the Double-Layer Properties of Gold Faces in Perchloric Acid Solutions: Part I. The (210) Gold Face. *J. Electroanal. Chem. Interfacial Electrochem.* **1987**. https://doi.org/10.1016/0022-0728(87)85134-3.

(4) Valette, G. Double Layer on Silver Single Crystal Electrodes in Contact with Electrolytes Having Anions Which Are Slightly Specifically Adsorbed: Part III. The (111) Face. *J. Electroanal. Chem. Interfacial Electrochem.* **1989**, *269* (1), 191–203. https://doi.org/10.1016/0022-0728(89)80112-3.

(5) Valette, G. Double Layer on Silver Single-Crystal Electrodes in Contact with Electrolytes Having Anions Which Present a Slight Specific Adsorption PART I. THE (110) FACE. *J. Electroanal. Chem. Interfacial Electrochem.* **1981**, *122*, 285–297. https://doi.org/10.1016/S0022-0728(81)80159-3.

(6) Valette, G. Double Layer on Silver Single Crystal Electrodes in Contact with Electrolytes Having Anions Which Are Slightly Specifically Adsorbed: Part II. The (100) Face. *J. Electroanal. Chem. Interfacial Electrochem.* **1982**, *138* (1), 37–54. https://doi.org/10.1016/0022-0728(82)87126-X.

(7) Tang, W.; Zhao, S.; Huang, J. Origin of Solvent Dependency of the Potential of Zero Charge. *JACS Au* **2023**, *3* (12), 3381–3390. https://doi.org/10.1021/jacsau.3c00552.

(8) Zhang, Z.; Huang, J. Microstructure of Electrical Double Layers at Highly Charged States. *JACS Au* **2025**. https://doi.org/10.1021/jacsau.5c00508.

(9) Valette, G. Hydrophilicity of Metal Surfaces: Silver, Gold and Copper Electrodes. *J. Electroanal. Chem. Interfacial Electrochem.* **1982**, *139* (2), 285–301. https://doi.org/10.1016/0022-0728(82)85127-9.

(10) Schnur, S.; Groß, A. Properties of Metal–Water Interfaces Studied from First Principles. *New J. Phys.* **2009**, *11* (12), 125003. https://doi.org/10.1088/1367-2630/11/12/125003.

(11) Zhen, E.; Chen, Y.; Huang, J. Double-Layer Capacitance Peaks: Origins, Ion Dependence, and Temperature Effects. *J. Chem. Phys.* **2025**, *162* (14), 144702. https://doi.org/10.1063/5.0251548.

(12) Gerischer, H. An Interpretation of the Double Layer Capacity of Graphite Electrodes in Relation to the Density of States at the Fermi Level. *J. Phys. Chem.* **1985**, *89* (20), 4249–4251. https://doi.org/10.1021/j100266a020.

(13) Gerischer, H.; McIntyre, R.; Scherson, D.; Storck, W. Density of the Electronic States of Graphite: Derivation from Differential Capacitance Measurements. *J. Phys. Chem.* **1987**, *91* (7), 1931–1935. https://doi.org/10.1021/j100291a049.

(14) Luque, N. B.; Schmickler, W. The Electric Double Layer on Graphite. *Electrochimica Acta* **2012**, *71*, 82–85. https://doi.org/10.1016/j.electacta.2012.03.083.

(15) Climent, V.; Gómez, R.; Feliu, J. M. Effect of Increasing Amount of Steps on the Potential of Zero Total Charge of Pt(111) Electrodes. *Electrochimica Acta* **1999**, *45* (4), 629–637. https://doi.org/10.1016/S0013-4686(99)00241-8.

(16) Gómez, R.; Climent, V.; Feliu, J. M.; Weaver, M. J. Dependence of the Potential of Zero Charge of Stepped Platinum (111) Electrodes on the Oriented Step-Edge Density: Electrochemical Implications and Comparison with Work Function Behavior. *J. Phys. Chem. B* **2000**, *104* (3), 597–605. https://doi.org/10.1021/jp992870c.

(17) Climent, V.; Attard, G. A.; Feliu, J. M. Potential of Zero Charge of Platinum Stepped Surfaces: A Combined Approach of CO Charge Displacement and N2O Reduction. *J. Electroanal. Chem.* **2002**, *532* (1), 67–74. https://doi.org/10.1016/S0022-0728(02)00849-5.

(18) Huang, J. Density-Potential Functional Theory of Electrochemical Double Layers: Calibration on the Ag(111)-KPF $_6$ System and Parametric Analysis. *J. Chem. Theory Comput.* **2023**, *19* (3), 1003–1013. https://doi.org/10.1021/acs.jctc.2c00799.

(19) Grahame, D. C. The Electrical Double Layer and the Theory of Electrocapillarity. *Chem. Rev.* **1947**, *41* (3), 441–501. https://doi.org/10.1021/cr60130a002.

(20) Zhang, M.; Chen, Y.; Eikerling, M.; Huang, J. Structured Solvent on a Split Electron Tail: A Semiclassical Theory of Electrified Metal-Solution Interfaces. *Phys. Rev. Appl.* **2025**, *23* (2), 24009. https://doi.org/10.1103/PhysRevApplied.23.024009.

(21) Perdew, J. P.; Kurth, S. Density Functionals for Non-Relativistic Coulomb Systems in the New Century. In *A Primer in Density Functional Theory*; Fiolhais, C., Nogueira, F., Marques, M. A. L., Eds.; Springer: Berlin, Heidelberg, 2003; pp 1–55. https://doi.org/10.1007/3-540-37072-2_1.
(22) Lundqvist, S.; March, N. H. *Theory of the Inhomogeneous Electron Gas*; Springer Science & Business Media, 2013.
(23) Constantin, L. A.; Fabiano, E.; Della Sala, F. Performance of Semilocal Kinetic Energy Functionals for Orbital-Free Density Functional Theory. *J. Chem. Theory Comput.* **2019**, *15* (5), 3044–3055. https://doi.org/10.1021/acs.jctc.9b00183.
(24) Johnson, P. B.; Christy, R. W. Optical Constants of the Noble Metals. *Phys. Rev. B* **1972**, *6* (12), 4370–4379. https://doi.org/10.1103/PhysRevB.6.4370.
(25) Kuznetsov, A. M.; Ulstrup, J. *Electron Transfer in Chemistry and Biology: An Introduction to the Theory*; John Wiley & Sons Ltd: Chichester, UK, 1999.
(26) Bikerman, J. J. XXXIX. Structure and Capacity of Electrical Double Layer. *Lond. Edinb. Dublin Philos. Mag. J. Sci.* **1942**, *33* (220), 384–397. https://doi.org/10.1080/14786444208520813.
(27) Freise, V. Zur Theorie Der Diffusen Doppelschicht. *Z. Für Elektrochem. Berichte Bunsenges. Für Phys. Chem.* **1952**, *56* (8), 822–827. https://doi.org/10.1002/bbpc.19520560826.
(28) Eigen, M.; Wicke, E. The Thermodynamics of Electrolytes at Higher Concentration. *J. Phys. Chem.* **1954**, *58* (9), 702–714. https://doi.org/10.1021/j150519a007.
(29) Le, J.; Cuesta, A.; Cheng, J. The Structure of Metal-Water Interface at the Potential of Zero Charge from Density Functional Theory-Based Molecular Dynamics. *J. Electroanal. Chem.* **2018**, *819*, 87–94. https://doi.org/10.1016/j.jelechem.2017.09.002.
(30) Schmickler, W. A Jellium-Dipole Model for the Double Layer. *J. Electroanal. Chem. Interfacial Electrochem.* **1983**, *150* (1), 19–24. https://doi.org/10.1016/S0022-0728(83)80185-5.
(31) Badiali, J. P.; Rosinberg, M. L.; Vericat, F.; Blum, L. A Microscopic Model for the Liquid Metal-Ionic Solution Interface. *J. Electroanal. Chem. Interfacial Electrochem.* **1983**, *158* (2), 253–267. https://doi.org/10.1016/S0022-0728(83)80611-1.
(32) Schmickler, W.; Henderson, D. The Interphase between Jellium and a Hard Sphere Electrolyte. A Model for the Electric Double Layer. *J. Chem. Phys.* **1984**, *80* (7), 3381–3386. https://doi.org/10.1063/1.447092.
(33) Kornyshev, A. A. Metal Electrons in the Double Layer Theory. *Electrochimica Acta* **1989**, *34* (12), 1829–1847. https://doi.org/10.1016/0013-4686(89)85070-4.
(34) Borukhov, I.; Andelman, D.; Orland, H. Steric Effects in Electrolytes: A Modified Poisson-Boltzmann Equation. *Phys. Rev. Lett.* **1997**, *79* (3), 435–438. https://doi.org/10.1103/PhysRevLett.79.435.
(35) Gongadze, E.; Iglič, A. Decrease of Permittivity of an Electrolyte Solution near a Charged Surface Due to Saturation and Excluded Volume Effects. *Bioelectrochemistry* **2012**, *87*, 199–203. https://doi.org/10.1016/j.bioelechem.2011.12.001.
(36) Gongadze, E.; Van Rienen, U.; Kralj-Iglič, V.; Iglič, A. Spatial Variation of Permittivity of an Electrolyte Solution in Contact with a Charged Metal Surface: A Mini Review. *Comput. Methods Biomech. Biomed. Engin.* **2013**, *16* (5), 463–480. https://doi.org/10.1080/10255842.2011.624769.
(37) Wicke, E.; Eigen, M. Über Den Einfluß Des Raumbedarfs von Ionen in Wäßriger Lösung Auf Ihre Verteilung in Elektrischen Feld Und Ihre Aktivitätskoeffizienten. *Z. Für Elektrochem. Berichte Bunsenges. Für Phys. Chem.* **1952**, *56* (6), 551–561. https://doi.org/10.1002/bbpc.19520560607.
(38) Yu, Y.-X.; Wu, J. Structures of Hard-Sphere Fluids from a Modified Fundamental-Measure Theory. *J. Chem. Phys.* **2002**, *117* (22), 10156–10164. https://doi.org/10.1063/1.1520530.
(39) Huang, J. Hybrid Density-Potential Functional Theory of Electric Double Layers. *Electrochimica Acta* **2021**, *389*, 138720. https://doi.org/10.1016/j.electacta.2021.138720.
(40) Cheng, J.; Sprik, M. Alignment of Electronic Energy Levels at Electrochemical Interfaces. *Phys. Chem. Chem. Phys.* **2012**, *14* (32), 11245–11267. https://doi.org/10.1039/C2CP41652B.
(41) Le, J.-B.; Cheng, J. Modeling Electrochemical Interfaces from Ab Initio Molecular Dynamics: Water Adsorption on Metal Surfaces at Potential of Zero Charge. *Curr. Opin. Electrochem.* **2020**, *19*, 129–136. https://doi.org/10.1016/j.coelec.2019.11.008.
(42) Trasatti, S. Interfacial Behaviour of Non-Aqueous Solvents. *Electrochimica Acta* **1987**, *32* (6), 843–850. https://doi.org/10.1016/0013-4686(87)87072-X.

(43) Gongadze, E.; Velikonja, A.; Perutkova, Š.; Kramar, P.; Maček-Lebar, A.; Kralj-Iglič, V.; Iglič, A. Ions and Water Molecules in an Electrolyte Solution in Contact with Charged and Dipolar Surfaces. *Electrochimica Acta* **2014**, *126*, 42–60. https://doi.org/10.1016/j.electacta.2013.07.147.
(44) Huang, J.; Chen, S.; Eikerling, M. Grand-Canonical Model of Electrochemical Double Layers from a Hybrid Density–Potential Functional. *J. Chem. Theory Comput.* **2021**, *17* (4), 2417–2430. https://doi.org/10.1021/acs.jctc.1c00098.
(45) Böcker, J.; Gurskii, Z.; Heinzinger, K. Structure and Dynamics at the Liquid Mercury–Water Interface. *J. Phys. Chem.* **1996**, *100* (36), 14969–14977. https://doi.org/10.1021/jp961065k.
(46) Sellers, H.; Sudhakar, P. V. The Interaction between Water and the Liquid-Mercury Surface. *J. Chem. Phys.* **1992**, *97* (9), 6644–6648. https://doi.org/10.1063/1.463668.
(47) Huang, J.; Domínguez-Flores, F.; Melander, M. Variants of Surface Charges and Capacitances in Electrocatalysis: Insights from Density-Potential Functional Theory Embedded with an Implicit Chemisorption Model. *PRX Energy* **2024**, *3* (4), 43008. https://doi.org/10.1103/PRXEnergy.3.043008.
(48) Tang, W.; Zhao, S.; Eikerling, M.; Huang, J. Low Parsons–Zobel Slope of Metal–Solution Interfaces: A Theoretical Comparison of Three Causes. *AIChE J.* **2025**, e70133. https://doi.org/10.1002/aic.70133.
